# EZH2 inhibitor SHR2554 enhances the anti-tumor efficacy of HDAC inhibitor Chidamide through STAT1 in T-cell lymphoma

**DOI:** 10.1038/s41419-025-07775-x

**Published:** 2025-07-14

**Authors:** Jiajin Wu, Dingyao Hu, Hui Yu, Dedao Wang, Yingying Ye, Jiaowu Cao, Tao Pan, Lan Mi, Yuqin Song, Meng Wu, Lingyan Ping, Jun Zhu

**Affiliations:** Key Laboratory of Carcinogenesis and Translational Research (Ministry of Education), Department of Lymphoma, Peking University Cancer Hospital & Institute, Beijing, 100142 China

**Keywords:** Non-hodgkin lymphoma, Cancer epigenetics

## Abstract

T-cell lymphoma (TCL) is a rare subtype of non-Hodgkin lymphoma (NHL) that is associated with a poor prognosis. Although HDAC inhibitors have been approved for TCL treatment for several years, their expected therapeutic efficacy remains unmet in some patients. In this study, we discovered that TCL tumor cells develop resistance to HDAC inhibitor treatment by upregulating the methylation of lysine 27 on histone H3 (H3K27me3) levels. Furthermore, we confirmed the pharmacological efficacy of the EZH2 inhibitor SHR2554 and demonstrated a synergistic effect when combined with the HDAC inhibitor Chidamide through commercial TCL cell lines, in vivo cell-derived xenograft, and patient-derived xenograft cancer models. We inferred that STAT1 was the key driver of the synergistic effect using RNA-seq and ChIP-seq analysis. Our findings provide sufficient preclinical evidence in support of a potential combination therapy strategy for TCL patients.

## Introduction

T-cell lymphoma (TCL) consists of a group of heterogeneous hematological malignancies that continue to have a poor prognosis. Existing therapeutic regimens for TCL are primarily adapted from those used in B-cell lymphoma, including CHOP and CHOPE. However, they haven’t been as effective as predicted in some cases. The 5-year overall survival (OS) rates for common subtypes have not significantly improved over the past 20 years [1]. Therefore, there is an urgent need for in-depth research on therapeutic strategies.

Epigenetic dysregulation has been associated with cancer metastasis and progression [2]. Histone deacetylases (HDAC) and histone acetyltransferases (HAT) regulate the homeostasis of histone acetylation, thereby serving as critical epigenetic regulators in the pathobiology of various malignancies, including lymphoma [3, 4]. Abnormalities in HDAC activity disrupt the equilibrium between acetylation and deacetylation, resulting in a more condensed nucleosome structure that downregulates tumor suppressor genes and subsequently promotes carcinogenesis [5, 6]. HDAC inhibitors have been approved by the Food and Drug Administration for the treatment of TCL for several years [7]. Chidamide was approved in China in 2014 for the treatment of relapsed or refractory PTCL, with an overall response rate (ORR) of 28% [8]. However, in patients with TCL, treatment with these drugs resulted in an ORR that often fell below 35% [9, 10]. It is noteworthy that HDAC inhibitors have demonstrated considerable utility when combined with other small-molecule drugs to enhance therapeutic efficacy [11].

EZH2 functions as a critical epigenetic regulator, catalyzing the trimethylation of lysine 27 on histone H3 (H3K27me3) and thereby silencing downstream target genes through a classical mechanism. Overexpression of EZH2 has been reported in several human malignancies, leading to EZH2 dysfunction and the suppression of tumor suppressor gene expression in conditions such as prostate cancer, NK/T-cell lymphoma, and melanoma [12–14]. EZH2 acts as a transcriptional coactivator in the MYCN-mediated gene expression program in TCL [15]. Several EZH2 inhibitors have already been in clinical studies [16–18]. Clinical studies on the EZH1/2 dual-target inhibitor valemetostat are ongoing in adult T-cell leukemia/lymphoma (ATLL) [19].

HDAC and EZH2 regulate the balance between the acetylation and methylation of H3K27, thereby modulating gene expression. In this study, we found that tumor cells increase their levels of H3K27me3 to confer resistance to HDAC inhibitor treatment in TCL. The small-molecule inhibitor SHR2554, which targets EZH2, demonstrated significant anti-tumor effects on various TCL cell lines. The combination of EZH2 inhibitors with HDAC inhibitors in the treatment of TCL demonstrated a synergistic effect both in vitro and in mouse models. EZH2 inhibitors counteract the H3K27me3 levels induced by HDAC inhibitors, thereby releasing tumor suppressor genes, including STAT1. These findings underscore the epigenetic relationship between H3K27ac and H3K27me3 in TCL, highlighting potential therapeutic strategies for TCL.

## Materials and Methods

### Cell lines and reagents

Available in supplementary material and methods.

### Cell viability assay and combination index (CI) analysis

Cells were seeded in 96-well plates at a density of 4 × 10^3^ cells per 100 μL per well and treated with the specified drugs at varying concentrations. Cell viability was assessed using the CellTiter-Glo Luminescent Cell Viability Assay System (Promega, Madison, USA, #G7572). Synergistic effects were analyzed using CompuSyn software (ComboSyn Inc, NJ, USA) [20].

### Cell apoptosis and cell cycle assays

For the apoptosis assay, cells were resuspended in 1× Binding Buffer and stained with Annexin V-FITC and propidium iodide (PI) using an apoptosis detection kit (Dojindo Laboratories, Kumamoto, Japan, #AD10). For cell cycle analysis, cells were harvested and fixed in pre-cooled 75% ethanol at −20 °C and stored at 4 °C overnight. The following day, the fixed cells were washed with PBS and subsequently resuspended in PI/RNase solution (KeyGen BioTECH, Jiangsu, China, #KGA511) for staining at room temperature for 30 to 60 min. Finally, all samples were analyzed using the BECKMAN CytoFLEX (BECKMAN COULTER Life Sciences, Indiana, USA).

### RNA extraction, real-time PCR, immunohistochemistry (IHC), and western blot analysis

Available in supplementary material and methods.

### In vivo study

All mouse experiments were conducted in accordance with the guidelines set forth by the Institutional Animal Care and Use Committee of Peking University Cancer Hospital & Institute and adhered to the principles outlined in the Guide for the Care and Use of Laboratory Animals [21]. Female immune-deficient NOD.Cg-Prkdc^scidIl2rg^tm1Sug/Jic (NCG) mice, aged 5–6 weeks, were sourced from Beijing HFK Bioscience (Beijing, China) and were maintained in a specific pathogen-free environment within our facility. Tumor cells (H9, at 5 × 10^6^ cells) were suspended in an equal volume of PBS and Matrigel (Corning, NY, USA, #356237) and subsequently inoculated subcutaneously into the right flank of each mouse. For the patient-derived xenograft (PDX) model, a 1 mm³ biopsy specimen from a patient was subcutaneously injected into the right side of each mouse. SHR2554 and Chidamide were prepared according to the in vivo experimental protocol from MCE, using a solvent mixture of 10% DMSO, 40% PEG300, 5% Tween-80, and 45% saline. Tumors measuring 150–200 mm³ will be assigned randomly into groups. Body weight and tumor volume (V) of the mice were assessed every two days, with tumor volume calculated using the formula V = (length × width²)/2 (mm³) by a digital caliper. Mice were ultimately sacrificed when body weight loss exceeded 20% or when tumor volume surpassed 1500–2000 mm^3^.

### RNA-seq, ChIP-seq, lentivirus packing and infection, and statistical analysis

Available in supplementary material and methods.

## Results

### TCL tumor cells with acquired resistance to HDAC inhibitors exhibited elevated H3K27me3

**Fig. 1:**
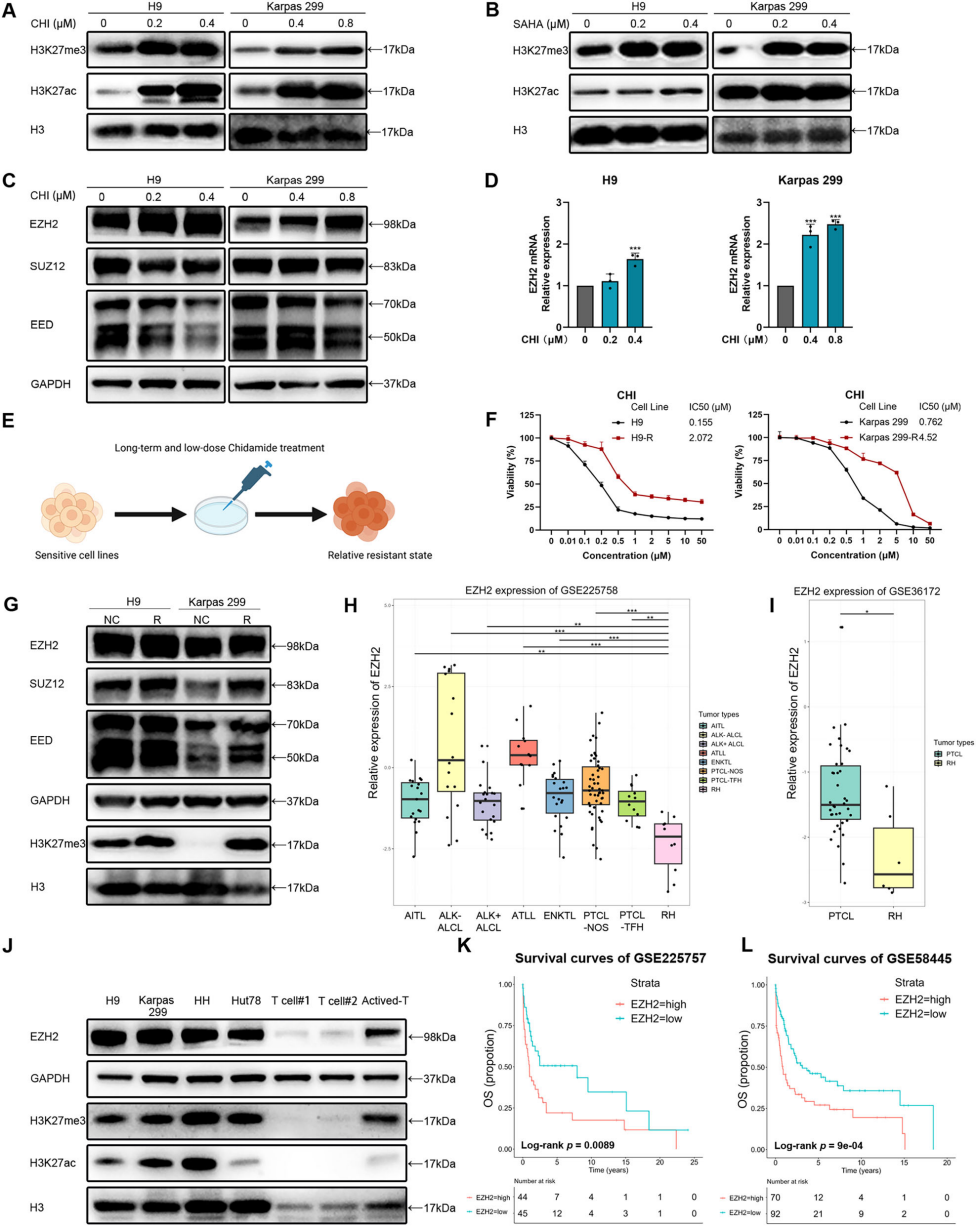
TCL tumor cells with acquired resistance to HDAC inhibitors exhibited elevated H3K27me3. **A**, **B** Western blot analysis showed increased levels of H3K27me3 and H3K27ac following treatment with HDAC inhibitors Chidamide (**A**) and SAHA (**B**) in the H9 and Karpas 299 cell lines at the indicated concentrations for 48 h (1 × 10^5^ cells/mL), compared to the DMSO control. Histone H3 (H3) was used as a loading control. **C** Western blot analysis was performed to assess the protein levels of the core subunits of the PRC2 complex in the H9 and Karpas 299 cell lines, including EZH2, SUZ12, and EED, following treatment with CHI. GAPDH was used as a loading control. **D** RT-PCR was performed to assess mRNA expression of EZH2, following treatment with CHI. GAPDH was used as a loading control. **E** Schematic diagram of the process for inducing drug-resistant cell lines. Drug-resistant TCL cell lines were induced through prolonged treatment with 20% inhibitory concentration (IC20). **F** Dose-response curves for CHI were generated comparing drug-resistant cell lines (R) with control cell lines (NC). CHI was diluted from an initial concentration of 50 μM and treated at a density of 4 × 10^3^ cells/mL/well for 48 h. The 48-hour 50% inhibitory concentration (IC50) values were calculated using SPSS and are presented accordingly. **G** Western blot analysis was performed to assess the variations in protein levels of EZH2, SUZ12, EED, and H3K27me3 between drug-resistant cell lines (R) and control cell lines (NC). GAPDH or H3 was used as a loading control. **H**, **I** Gene expression profiling analysis from GSE225758 (**H**) and GSE36172 (**I**) demonstrated significantly elevated mRNA levels of EZH2 in patients with TCL compared to those with reactive hyperplasia of lymph nodes (RH). **J** Western blot analysis was performed to assess variations in the protein levels of EZH2, H3K27me3, and H3K27ac among TCL cell lines and T cells. GAPDH or H3 was used as a loading control. **K**, **L** Kaplan–Meier curves for OS of TCL patients based on EZH2 mRNA expression from GSE225757 (**K**) and GSE58445 (**L**). A Log-Rank test was performed to assess statistical differences between the two groups. Tick marks indicate censored data. CHI, Chidamide. SAHA, Vorinostat. AITL, Angioimmunoblastic T-cell lymphoma. ALCL, Anaplastic large cell lymphoma. ATLL, Adult T-cell leukemia/lymphoma. ENKTL, Extranodal Natural Killer/T-cell Lymphoma. PTCL, peripheral T cell lymphoma. RH, reactive hyperplasia. OS, Overall survival. All experiments were conducted in triplicate, and the data are reported as mean ± SD. *p < 0.05, **p < 0.01, ***p < 0.001, compared with the control group.

To investigate the resistance mechanism of TCL tumor cells to HDAC inhibitors, we treated four TCL cell lines with Chidamide (CHI) for 48 h. These cell lines encompass different types of TCL, including cutaneous T-cell lymphoma (CTCL), anaplastic large T-cell lymphoma (ALCL), and ATLL. The tumor cell lines exhibited a dose-dependent increase in H3K27ac and H3K27me3 levels following CHI treatment. (Fig. 1A and Supplementary Fig. S1A, B). The increase in H3K27ac levels is induced by HDAC inhibition, whereas elevated levels of H3K27me3 lead to the silencing of tumor suppressor genes, thereby promoting tumor development. This is in contrast to the gene activation driven by increased H3K27ac levels [22, 23]. Therefore, we hypothesize that the increase in H3K27me3 levels leads to the silencing of specific tumor suppressor genes. To confirm that HDAC inhibitors increase H3K27me3 levels, we utilized another HDAC inhibitor, Vorinostat (SAHA), in these four cell lines. Consistent with CHI, SAHA significantly elevated H3K27me3 levels in the cancer cell lines (Fig. 1B and Supplementary Fig. S1C, D). H3K27me3 is mediated by PRC2, which comprises core subunits, including EZH2, SUZ12, and EED [24]. The immunoblot results indicated that increasing concentrations of CHI led to elevated expression of EZH2 in all four cell lines, rather than SUZ12 or EED (Fig. 1C and Supplementary Fig. S1E, F). CHI and SAHA block HDAC enzymatic activity through competitive binding without promoting the direct degradation of HDAC proteins. In TCL cell lines, the expression levels of their common molecular targets (HDAC1, HDAC2, HDAC3) remained unchanged, consistent with their non-degradative mechanism of action (Supplementary Fig. S2A, B). Additionally, we evaluated the mRNA expression levels of these three subunits following CHI treatment. We found that the mRNA expression of EZH2 was dose-dependently upregulated in the CTCL cell line H9 and the ALCL cell line Karpas 299 (Fig. 1D and Supplementary Fig. S2C). We further induced the tumor cell lines with long-term, low-dose HDAC inhibitor treatment to establish resistant cell strains H9-R, K299-R, and HUT78-R (Fig. 1E, F and Supplementary Fig. S2D). The resistant cell lines exhibited a significant upregulation of H3K27me3 levels compared to the control cell lines (Fig. 1G and Supplementary Fig. S2E). The observed transcriptomic and protein alterations suggest that TCL tumor cells upregulate H3K27me3 primarily through EZH2 as a mechanism to resist HDAC inhibitors.

Subsequently, we investigated the transcriptome of EZH2 in TCL patients. Compared to the mRNA expression levels in reactive hyperplasia lymph nodes from the GSE225757 and GSE225758 datasets, EZH2 was significantly upregulated across all subtypes of TCL (Fig. 1H and Supplementary Fig. S2F). Additionally, an increase in EZH2 mRNA levels was observed in PTCL compared to reactive lymph nodes in the GSE36172 dataset (Fig. 1I). To validate the elevated expression of EZH2 protein in TCL, we compared the EZH2 expression levels between tumor cell lines and normal T cells. Our findings demonstrated that the protein expression of EZH2 in tumor cell lines was significantly higher than that in normal T cells, accompanied by increased levels of H3K27me3. Furthermore, we showed that upon activation, T cells exhibited elevated levels of EZH2 and H3K27me3 (Fig. 1J). The survival analysis of datasets GSE225757 and GSE58445 further indicated that TCL patients exhibiting high expression levels of EZH2 demonstrated significantly poorer overall survival (OS) rates (Fig. 1K, L). We subsequently evaluated 16 patients with angioimmunoblastic T cell lymphoma (AITL) who received the CHOPE regimen at Peking University Cancer Hospital and found that individuals with elevated EZH2 expression exhibited reduced progression-free survival (PFS) (Supplementary Fig. S2G). The transcriptomic anomalies identified suggest that EZH2 may play a crucial role in the progression of TCL. These results indicate that EZH2, as an oncogene, is activated following HDAC inhibitor treatment and may contribute to the resistance of TCL tumor cells to HDAC inhibitors.

### The EZH2 inhibitor SHR2554 mediates therapeutic effects in TCL by reducing H3K27me3 levels

**Fig. 2:**
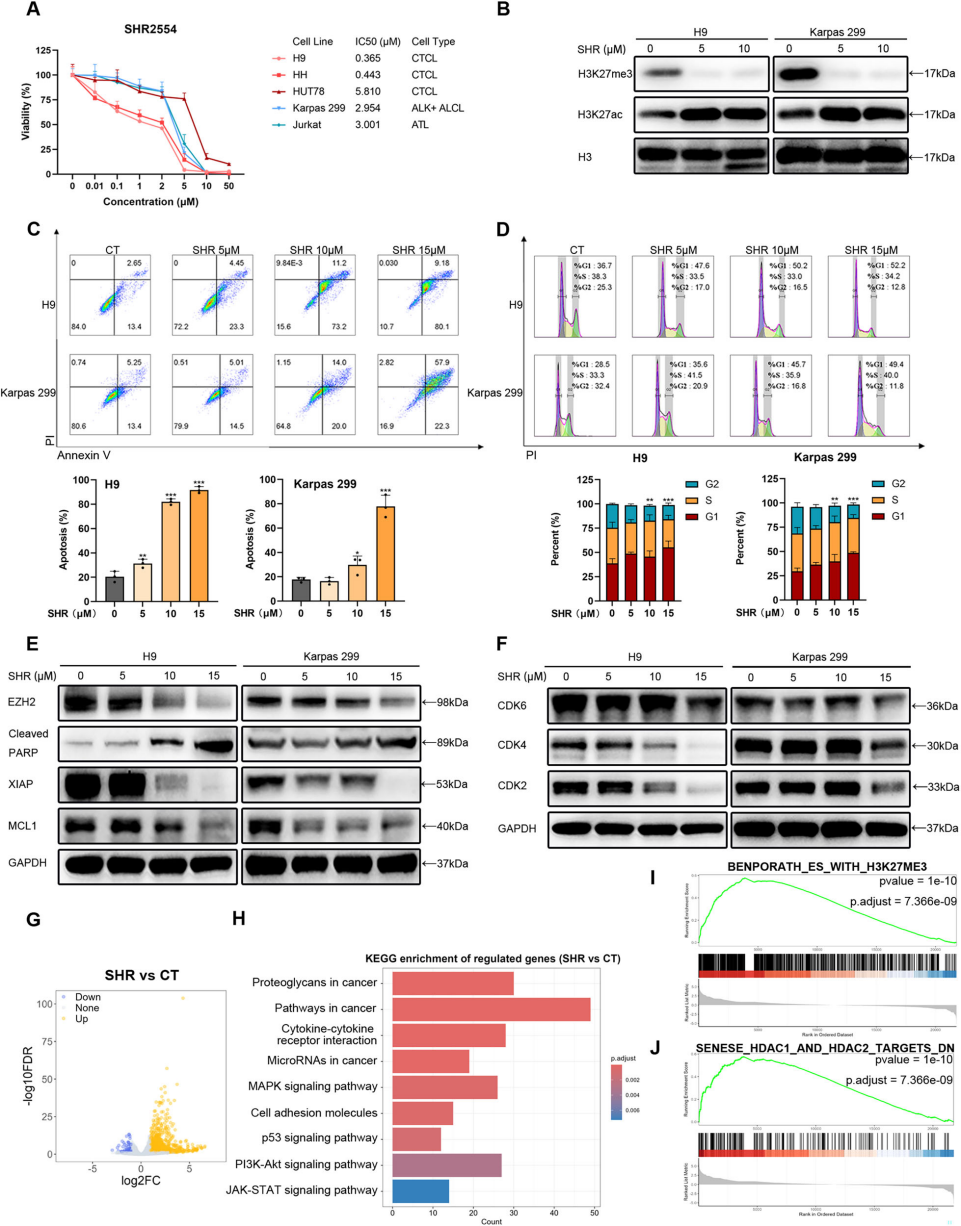
The EZH2 inhibitor SHR2554 mediates therapeutic effects in TCL by reducing H3K27me3 levels. **A** Dose-response curves for SHR were generated across 5 TCL cell lines. SHR was diluted from an initial concentration of 50 μM and treated at a density of 4 × 10^3^ cells/mL/well for 144 h. The control group received the same concentration of DMSO. The 144-hour 50% inhibitory concentration (IC50) values were calculated using SPSS and are presented accordingly. **B** Western blot analysis was performed to assess the levels of H3K27me3 and H3K27ac following treatment with SHR for 72 h. H3 was used as a loading control. **C**, **D** 1 × 10^5^ cells/mL were cultured in the presence of the indicated concentrations of SHR for 72 h. Cell apoptosis (**C**) and cell cycle (**D**) were assessed by flow cytometry. **E**, **F** Western blot analysis was performed to assess cell apoptosis (**E**) and cell cycle-related proteins (**F**). GAPDH was used as a loading control. **G** Volcano plot illustrating the changes in gene expression in the SHR group (SHR 5 μM, 72 h) compared to the control group in RNA-seq. **H** KEGG enrichment of regulated genes in the SHR group compared to the control group. **I**, **J** Gene set enrichment (GSEA) plot of BENPORATH_ES_WITH_H3K27ME3 (**I**) and SENESE_HDAC1_AND_HDAC2_TARGETS_DN (**J**) gene sets comparing SHR and the control group. SHR, SHR2554. All experiments were conducted in triplicate., and data are reported as mean ± SD. Experimental groups were compared to the DMSO control using One-Way ANOVA followed by LSD post hoc multiple comparisons. *p < 0.05, **p < 0.01, ***p < 0.001, compared with the control group.

To investigate the anti-tumor efficacy of targeting EZH2 in TCL, we conducted a cell viability assay on tumor cell lines treated with the EZH1/2 inhibitor Valemetostat, the EZH2 PROTAC degrader MS177, the EZH2 inhibitor EPZ6438 and SHR2554 (Supplementary Fig. S3A). Given the drug’s efficacy and its target, we selected SHR2554 (SHR) for further study. Treatment with SHR for 72 h and 144 h inhibited the proliferation of tumor cells in a dose-dependent manner, with IC50 values ranging from 0.365 to 3.001 μM for 144 h (Fig. 2A and Supplementary Fig. S3B). We confirmed that SHR reduced the levels of H3K27me3 in TCL and decreased EZH2 expression at high concentrations (Fig. 2B, E, and Supplementary Fig. S3C, D). We performed analysis of cell apoptosis and cell cycle profiles in TCL cells treated with SHR for 72 hours (Fig. 2C, D, and Supplementary Fig. S3D, E). Both the H9 and Karpas 299 cell lines exhibited a higher percentage of apoptotic cells in response to increasing doses of SHR (Fig. 2C). The levels of pro-apoptotic proteins, including cleaved PARP in its active form, were significantly elevated with increasing doses of SHR. Concurrently, a reduction in several anti-apoptotic proteins, including XIAP and MCL1, was observed (Fig. 2E and Supplementary Fig. S3D). Flow cytometry analysis indicated a notable increase in the percentage of cells in the G1/S phase following SHR treatment (Fig. 2D). In alignment with the flow cytometry data, proteins preferentially expressed in the G1 phase (CDK4, CDK6) and S phase (CDK2) were downregulated by SHR treatment (Fig. 2F and Supplementary Fig. S3E).

To investigate the targets of EZH2 in TCL, we performed transcriptome sequencing on the H9 cell line treated with SHR for 72 h. Consistent with the effect of inhibiting H3K27me3-modified gene expression, the number of upregulated genes was significantly greater than that of downregulated genes (Fig. 2G). The regulated genes were predominantly associated with the PI3K/AKT, MAPK, p53, and JAK-STAT signaling pathways, as revealed by our KEGG enrichment analysis (Fig. 2H). GO enrichment analysis indicated that RNA polymerase II is involved in gene regulation (Supplementary Fig. S3F). We performed GSEA analysis on all RNA-seq genes. In the SHR group, in addition to the upregulation of H3K27me3 target genes, genes downregulated by HDAC1, HDAC2, and HDAC3 were also upregulated, indicating the relationship between EZH2 and HDAC in epigenetic regulation (Fig. 2I, J and Supplementary Fig. S3G). We also conducted ChIP-seq analysis for H3K27me3 modifications in both the SHR group and the control group. In the SHR group, multiple tumor suppressor genes, including the p53 downstream gene PERP and the PI3K/AKT pathway inhibitor gene INPP4B, were identified among the genes downregulated by H3K27me3 modifications and elevated at the mRNA expression level (Supplementary Fig. S3H, I). Collectively, the EZH2 inhibitor SHR shows promise as a potential therapy for TCL, potentially associated with alterations in the methylation and acetylation of the H3K27 region.

### SHR2554 enhances the efficacy of Chidamide by neutralizing HDAC inhibitor-induced elevations in H3K27me3 levels

**Fig. 3:**
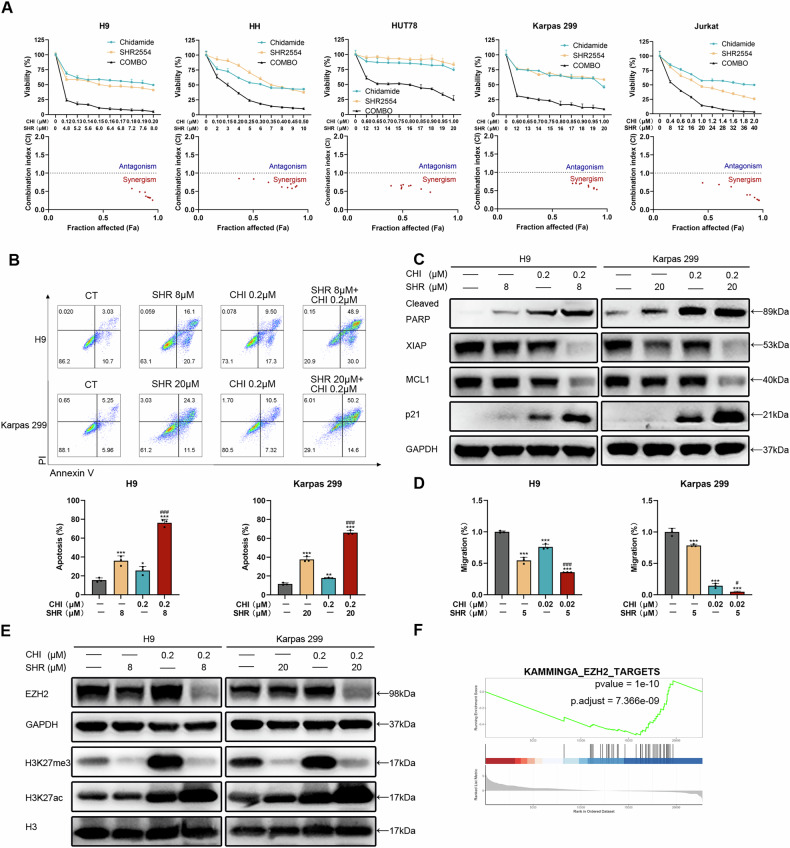
SHR2554 enhances the efficacy of Chidamide by neutralizing HDAC inhibitor-induced elevations in H3K27me3 levels. **A** Synergistic inhibitory effects of SHR and CHI on tumor cell proliferation. The concentrations of SHR and CHI were gradually increased in a fixed ratio from 0 according to their respective IC50 values. Combination index (CI) values were calculated using Compusyn and are presented at the bottom. CI values < 1 indicate a synergistic combination. **B** Representative flow cytometry images and histograms of the Annexin V for the cell apoptosis assay. Cell lines were treated with DMSO or the indicated concentrations for 48 h. **C** Western blot analysis was performed to assess cell apoptosis and cell cycle-related proteins. GAPDH was used as a loading control. **D** Representative histograms for the cell migration assay. Cell lines were treated with DMSO or the indicated concentrations for 48 h. **E** Western blot analysis was performed to assess variations in the protein levels of EZH2, H3K27me3, and H3K27ac. Cell lines were treated with DMSO or the indicated concentrations for 48 h. GAPDH or H3 was used as a loading control. **F** Gene set enrichment (GSEA) plot of KAMMINGA_EZH2_TARGETS gene set comparing between CHI and the control group. All experiments were conducted in triplicate, and data are reported as mean ± SD. Experimental groups were compared to the DMSO control using One-Way ANOVA followed by LSD post hoc multiple comparisons. *p < 0.05, **p < 0.01, ***p < 0.001, compared with the control group. #*p* < 0.05, ###*p* < 0.001 compared with CHI group.

Then, five TCL cell lines were treated with prescribed doses of the EZH2 inhibitor SHR2554 and the HDAC inhibitor Chidamide for 48 h. The combination therapy was administered at concentrations progressively increasing in a fixed ratio from 0, maintaining the IC50 values for SHR and CHI (Supplementary Fig. S4A). As illustrated in Fig. 3A, the synergistic inhibitory effect of the combination treatment was pronounced, as assessed by the CI value (CI < 1). Flow cytometry analysis and Western blot analysis revealed the combination of SHR and CHI induces a synergistic effect in triggering apoptosis, including the upregulation of cleaved PARP and the downregulation of XIAP and MCL1. Furthermore, we validated that p21, a tumor suppressor gene regulated by EZH2, exhibited a marked increase in expression when co-treated with the HDAC inhibitor (Fig. 3B, C and Supplementary Fig. S4B). The expression of cell cycle-related proteins was also downregulated under the combined treatment (Supplementary Fig. S4C). Additionally, the cell migration was inhibited with simultaneous exposure to SHR and CHI (Fig. 3D). We observed a substantial reduction in EZH2 protein expression in the combination group compared to the monotherapy group. We further explored the impact of drug combinations on H3K27 modification. We confirmed that the EZH2 inhibitor SHR2554 can significantly diminish the increase in H3K27me3 induced by the HDAC inhibitor Chidamide. Concurrently, it was observed that SHR synergistically upregulated the modification of H3K27ac in combination with CHI. (Fig. 3E and Supplementary Fig. S4D). No synergistic effects of SHR and CHI were observed at other histone modification sites, including H3K9me3, H3K9ac, H4K12ac, and H4K16ac (Supplementary Fig. S4E). The results indicate that the EZH2 inhibitor does not antagonize the acetylation effect of the HDAC inhibitor at the site of H3K27. These findings suggest that while HDAC inhibitors exert therapeutic effects on TCL through acetylation, tumor cells may resist pharmacological therapy by upregulating H3K27me3. EZH2 inhibitors can reverse the elevated levels of H3K27me3, thus re-regulating the target genes to enhance the therapeutic effect of HDAC inhibitors and produce a synergistic drug combination effect.

### SHR2554 and Chidamide exert a synergistic effect through upregulation of STAT1

**Fig. 4:**
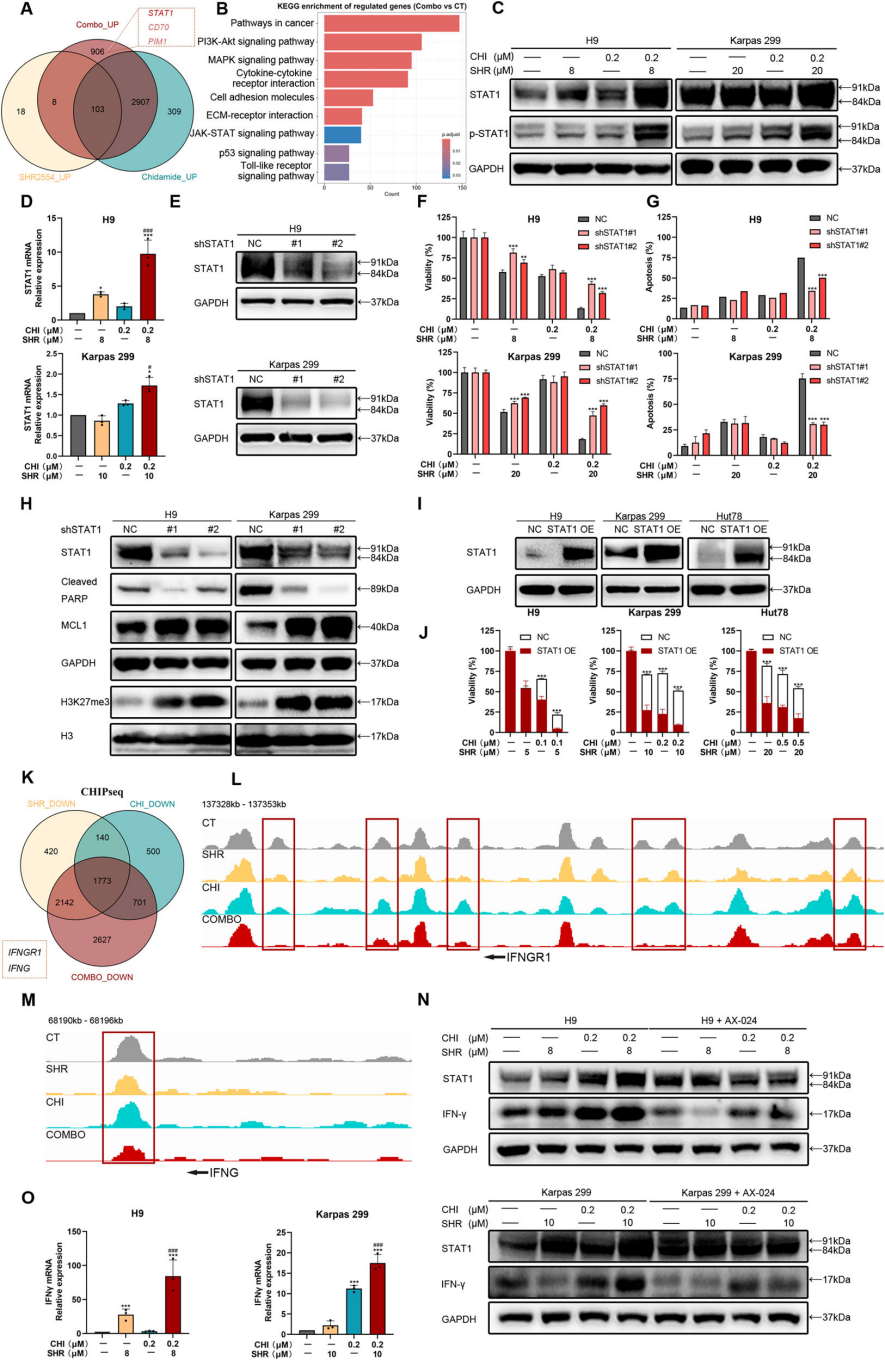
SHR2554 and Chidamide exert a synergistic effect through upregulation of STAT1. **A** Schematic representation of the overlapping upregulated genes (FC > 1.5, FDR < 0.05) from different treatment groups in RNA-seq (H9, SHR 8 μM, CHI 0.2 μM, SHR 8 μM + CHI 0.2 μM, 24 h, compared with DMSO control). **B** KEGG enrichment of regulated genes in the combo group compared to the control group from RNA-seq. **C**, **D** The protein (**C**) and mRNA (**D**) expression levels of STAT1 in H9 and Karpas 299 cells were analyzed after treatment with DMSO and the indicated concentrations of SHR and/or CHI for 48 h. GAPDH was used as a loading control. Representative figures are presented. **E** STAT1 knockdown by shRNA was validated by Western blot analysis. Cells were treated with 2 μg/mL doxycycline (Dox) for 48 h to induce stable STAT1 knockdown. GAPDH was used as a loading control. **F**, **G** The inhibition (**F**) and apoptosis (**G**) rates in STAT1 stably knockdown cell lines (NC, shSTAT1 #1, and shSTAT1 #2) were assessed following treatment with different groups. **H** Western blot analysis was performed to assess apoptosis-related proteins and H3K27 modifications in STAT1 stably knockdown cell lines. GAPDH or H3 was used as a loading control. **I** Western blot analysis was performed to validate the constructed STAT1 overexpression cell lines. **J** The inhibition (**F**) rates in STAT1 overexpression cell lines were assessed following treatment with different groups. **K** Venn diagram of H3K27me3 downregulated genes (Log2FC < -1, FDR < 0.05) from different treatment groups in ChIP-seq (H9, SHR 8 μM, CHI 0.2 μM, SHR 8 μM + CHI 0.2 μM, 48 h, compared with DMSO control). **L**, **M** IGV profiles demonstrating downregulated H3K27me3 occupancy on the IFNGR1 (**L**) and IFNG (**M**) in each treatment group. **N** Western blot analysis was performed to assess STAT1 and IFN-γ expression after treatment with the indicated concentrations of SHR and/or CHI and IFNγ inhibitor AX-024 (10 ng/ml) for 48 h. GAPDH was used as a loading control. **O** The mRNA expression levels of IFNG in H9 and Karpas 299 cells were analyzed after treatment with DMSO and the indicated concentrations of SHR and/or CHI for 48 h. GAPDH was used as a loading control. Representative figures are presented. All experiments were conducted in triplicate, and data are reported as mean ± SD. Experimental groups were compared to the DMSO control using One-Way ANOVA followed by LSD post hoc multiple comparisons. *p < 0.05, **p < 0.01, ***p < 0.001, compared with the control group. #*p* < 0.05, ###*p* < 0.001 compared with CHI group.

To explore the therapeutic mechanism associated with the combination of SHR2554 and Chidamide, we conducted transcriptome profiling of H9 cells under DMSO, SHR, CHI, and SHR + CHI treatments at 24 h. We initially investigated the reasons for the alterations in H3K27me3 levels under HDAC inhibitor treatments. Compared to the DMSO group, GSEA analysis of all genes in the CHI group indicated a significant downregulation of EZH2 target genes, which is consistent with the observed rise in H3K27me3 levels (Fig. 3F). Treatment with SHR for 24 hours resulted in fewer changes in gene expression compared to 72 hours, which is associated with the prolonged effect of H3K27me3 modifications (Supplementary Fig. S5A) [25]. A Venn diagram was used to illustrate the genes that were upregulated and downregulated in response to various treatments (FC > 1.5, FDR < 0.05). Compared to the DMSO group, the number of upregulated genes in each treatment group was significantly higher than that of downregulated genes, consistent with the observed variations in histone modifications post-treatment (Fig. 4A and Supplementary Fig. S5B). Our analysis focused on these upregulated genes, as they are more pertinent to the mechanisms of action of the drugs and histone modifications. In the combination group, the results of the KEGG enrichment analysis and GO enrichment analysis were substantially aligned with those obtained following treatment with EZH2 inhibitors, indicating the role of EZH2 inhibitors in combination therapy (Fig. 4B and Supplementary Fig. S5C). We noted that among the genes with high expression levels (FPKM > 30) in the combination group, the tumor suppressor gene STAT1 exhibited the highest expression (Fig. 4A).

The transcription factor STAT1 can regulate associated genes to limit tumor development and metastasis [26]. The protein levels of STAT1 also showed a significant increase in the H9 cell line following the combination treatment with the two drugs. However, in the Karpas 299 cell line, the activation of STAT1 was more evident in the increased phosphorylation levels, likely due to the exceptionally high basal expression of STAT1 in Karpas 299 (Fig. 4C and Supplementary Fig. S4D, E). Consistent with the protein results, qPCR analysis confirmed that the mRNA levels of STAT1 were markedly elevated following the combination of the two drugs, particularly in the H9 cell line (Fig. 4D). GSEA analysis revealed that genes containing STAT1 transcription factor binding sites were upregulated in the combination group (Supplementary Fig. S4F). Correlation analysis conducted across different databases consistently demonstrated a negative correlation between the expression levels of STAT1 and EZH2 (Supplementary Fig. S4G-I).

To gain a better understanding of the anti-tumor mechanisms underlying combination therapy and the role of STAT1 in TCL, we transfected STAT1 shRNA into H9 and Karpas 299 cells, confirming effective STAT1 knockdown via qPCR and Western blot analysis (Fig. 4E and Supplementary Fig. S6A, B). Cell viability assays indicated that the downregulation of STAT1 expression facilitated tumor cell proliferation (Supplementary Fig. S6C). After determining the drug concentrations from the combination assays in the STAT1 knockout cell lines, we observed that these knockout cell lines exhibited varying degrees of resistance to SHR and CHI, which significantly reduced the efficacy of the combination treatment (Fig. 4F). Flow cytometry analysis of apoptosis similarly revealed that, in the combination group of the STAT1 knockout cell lines, the percentage of apoptotic cells was substantially decreased, returning even to levels induced by single-agent treatment (Fig. 4G, Supplementary Fig. S6D, E). Western blot analysis confirmed a considerable decrease in cleaved-PARP and a notable increase in the anti-apoptotic protein MCL1 following STAT1 knockout. Interestingly, we also observed a significant increase in H3K27me3 modifications in the STAT1 knockout cell lines, while the expression of components of the PRC2 complex did not show significant changes (Fig. 4H and Supplementary Fig. S6F, G). The results indicate that while EZH2 modulates STAT1, it may also be subject to negative feedback regulation by STAT1. To further examine the role of STAT1 in combination therapy, we established STAT1 overexpression cell lines (Fig. 4I and Supplementary Fig. S6H). Due to the higher STAT1 expression levels in the Karpas 299 cell line, we also established another cell line, Hut78. In the STAT1 overexpressing cell lines, lower concentrations of SHR and CHI demonstrated excellent synergistic inhibition (Fig. 4J). The levels of H3K27me3 were relatively reduced. The expression of apoptotic proteins and the proliferation rate also exhibited an opposite pattern compared to those in the knockout cell lines (Supplementary Fig. S6I, J).

### The loss of H3K27me3 at the *IFNG* locus enhances IFN-γ expression and activates STAT1

To investigate the alterations in H3K27me3 modification with the combination of SHR2554 and Chidamide, we performed H3K27me3 ChIP-seq on H9 cells in each treatment group after 48 hours. Consistent with our findings of elevated genes from RNA-seq, we focused on the genes exhibiting reduced regions of H3K27me3 modifications. We discovered that the H3K27me3 modification of IFNGR1 was significantly downregulated in all treatment groups, whereas the H3K27me3 modification of IFNG was only significantly downregulated in the combination group (Fig. 4K). IFNG and IFNGR1 are upstream regulators of STAT1, and the binding of IFNG to IFNGR1 activates STAT1, exerting anti-tumor effects [27]. Meanwhile, in the combination group, the transcription levels of IFNG and IFNGR1 were significantly elevated (Supplementary Fig. S7A). Compared to IFNG, the reduction of H3K27me3 modification regions in IFNGR1 occurred more extensively and frequently (Fig. 4L, M and Supplementary Fig. S7B, C). Western blot analysis revealed a significant upregulation of IFN-γ expression under the combined treatment of the two drugs (Fig. 4N, Supplementary Fig. S7D). We also confirmed a significant elevation in IFNG mRNA expression via qPCR (Fig. 4O). The upregulation of IFN-γ expression is consistent with an increased expression of STAT1. Treatment with the IFN-γ inhibitor AX-024 not only suppressed IFN-γ expression but also abolished the upregulation of STAT1 induced by the combination therapy, suggesting that IFN-γ plays an activating role in STAT1 expression (Fig. 4N and Supplementary Fig. S7E) [28]. IFNG can bind to IFNGR1 to further enhance STAT1 expression, which may explain the considerable elevation of STAT1 observed primarily in the combination group [29]. Our results consistently indicate that SHR2554 enhances the efficacy of Chidamide by upregulating STAT1 through reducing H3K27me3 modification at the IFNG locus.

### Combination of SHR2554 and Chidamide exhibited potent antitumor effects in vivo

**Fig. 5:**
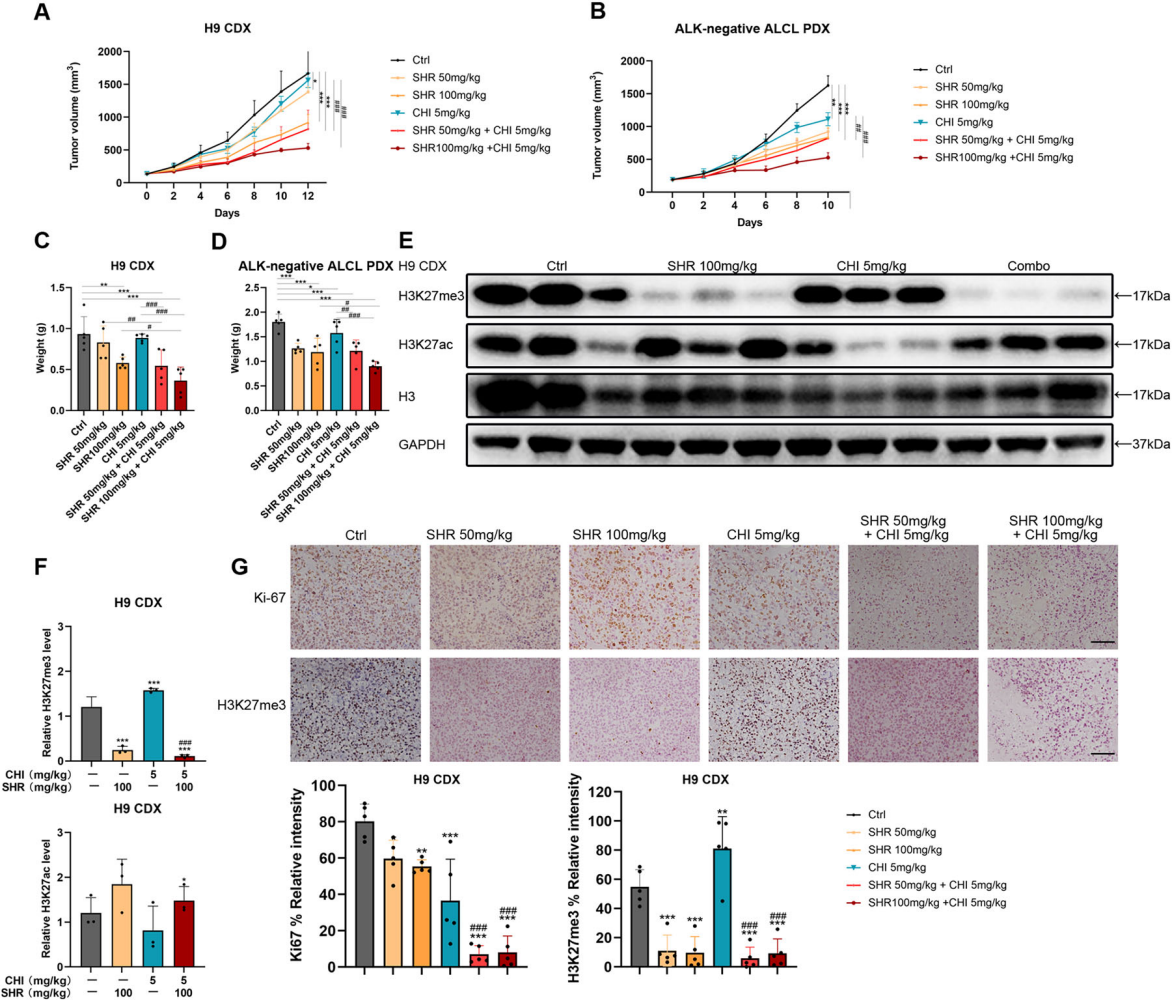
Combination of SHR2554 and Chidamide exhibited potent antitumor effects in vivo. **A**, **B** Efficacy of combination treatment in H9 CDX (**A**) and ALK-negative ALCL PDX (**B**) models. NCG mice bearing xenografts were treated daily by gavage with vehicle (grey), SHR (50 mg/kg and 100 mg/kg, yellow), CHI (5 mg/kg, blue), or the combination (red). The tumor growth curve illustrates changes in tumor volumes, measured every two days using calipers (mean ± SD, *n* = 5 for each group, V = (length × width²)/2 mm³). **C** and **D**. Tumor weights at the end of the experiment in different treatment groups of H9 CDX (C) and ALK-negative ALCL PDX (**D**) models. **E** Western blot analysis was performed to validate H3K27 modifications in vivo. H3 was used as a loading control. **F** Statistical data representing H3K27me3 and H3K27ac levels in H9 CDX models for each group. **G** Representative IHC analysis of Ki67 and H3K27me3 staining images of H9 CDX models. Statistics data represent of the positive cells of Ki67 and H3K27me3 for each group. All experiments were conducted in triplicate, and data are reported as mean ± SD. Experimental groups were compared to the DMSO control using One-Way ANOVA followed by LSD post hoc multiple comparisons. *p < 0.05, **p < 0.01, ***p < 0.001, compared with the control group. #*p* < 0.05, ##*p* < 0.01, ###*p* < 0.001 compared with CHI group.

In vivo experiments were conducted to further validate the therapeutic potential and safety of the combination strategy. We employed both cell line-derived xenograft (CDX) and patient-derived xenograft (PDX) models in the mouse experiments. This PDX model was derived from a biopsy specimen of a patient with ALK-negative ALCL. The pathological fidelity of this ALK-negative ALCL PDX was confirmed by positive staining for CD4, CD30, and Granzyme B, along with negative staining for ALK (Supplementary Fig. S7F). Once the tumor volume reached 150 mm^3^, all mice were randomized into six groups: control, SHR2554 (50 mg/kg and 100 mg/kg administered via gavage twice a day), Chidamide (5 mg/kg, administered via gavage daily), and a combination treatment group (*n* = 5 per treatment cohort). The administration of 5 mg/kg Chidamide daily alone resulted in only a modest reduction in tumor volume. However, when combined with varying concentrations of SHR2554, a significant decrease in tumor volume was observed, suggesting a promising synergistic effect between the two agents. The combination groups at high and low doses exhibited tumor inhibition rates of 50.9% and 68.1%, respectively, in H9 CDX (Fig. 5A, C). Moreover, the therapy was well tolerated, as demonstrated by the lack of weight loss or mortality in the mice (Supplementary Fig. S7G). Similarly, in the ALK-negative ALCL PDX model, the combination of the two drugs significantly improved therapeutic efficacy compared to single-agent treatment without weight loss (Fig. 5B, D, Supplementary Fig. S7H).

We further analyzed the modifications of H3K27 through Western blot analysis and immunohistochemical staining in H9 CDX. The Western blot and immunohistochemical staining results for H9 CDX protein confirmed consistent changes in H3K27me3 modification. We observed a significant increase in H3K27me3 in the CHI group, which was significantly inhibited upon combination with SHR. (Fig. 5E–G, Supplementary Fig. S7I). Additionally, Ki-67 staining of tumor sections revealed a significant decrease in proliferation, further confirming the inhibitory effect of the combination therapy on tumor growth (Fig. 5G). In summary, the combination of SHR2554 and Chidamide demonstrated promising anti-tumor effects in vivo in TCL, suggesting significant potential for clinical application.

## Discussion

Our study focuses on shifts in H3K27me3 modifications following pharmacological therapy and their associated effects on gene expression in TCL. Previous studies on the combination of HDAC inhibitors and EZH2 inhibitors in various cancers have largely overlooked the changes in H3K27me3 levels following HDAC inhibitor treatment [30**–**34]. In prostate cancer, both EZH2 and HDAC inhibitors collaboratively suppress the tumor suppressor function of ATF3 [31]. However, in our sequencing data, ATF3 is only upregulated following treatment with the EZH2 inhibitor (Supplementary Fig. S8A). In previous studies on HDAC inhibitors in B-cell lymphoma, alterations in H3K27me3 levels post-HDAC inhibitor treatment were not significant, which may explain the limited exploration of the combined mechanisms of HDAC inhibitors and EZH2 inhibitors from the perspective of H3K27me3 modifications [35].

In our study, we observed an aberrant elevation in H3K27me3 following the treatment of TCL with HDAC inhibitors. Previous patient sequencing data have indicated that EZH2 may lead to the aberrant activation of MYCN in PTCL, thereby contributing to tumor progression [15]. Our findings indicate that high EZH2 expression in patients is associated with decreased OS, consistent with observations in other malignancies [14, 36–41]. We conducted a series of experiments to evaluate the therapeutic efficacy of the combination of EZH2 inhibitors and HDAC inhibitors, as well as their underlying mechanisms. Our study reveals the substantial potential of EZH2 inhibitors in the treatment of TCL. We observed a relationship between EZH2 and HDAC in histone reprogramming. The application of EZH2 inhibitors facilitates the transition of the H3K27 locus from a methylated state to an acetylated modification, which typically results in the expression of target genes. This shift toward acetylation enhances H3K27ac modification following the administration of HDAC inhibitors, demonstrating the synergy between the two drugs. In various studies, the outcomes of gene transcription activation varied significantly based on the specific genes associated with elevated H3K27ac levels [30, 42–44]. One study indicated that the transition from methylation to acetylation can enhance Np63 expression and activate the p53 pathway, which is consistent with our findings [45, 46]. Additionally, our results suggest the aberrant activation of the MAPK pathway, which may also be a byproduct of the upregulation of H3K27ac (Supplementary Fig. S8B-D).

Our results suggest that HDAC inhibitors and EZH2 inhibitors generate synergistic effects by upregulating STAT1 through IFNG. Indeed, interferons were widely used in the treatment of TCL in the past [47]. IFNG is upstream of STAT1 and activates its anti-tumor effects by binding to IFNGR1 [48]. EZH2 governs life and death of peripheral T cells by inhibiting the secretion of IFNG [49]. In our study, the knockout of STAT1 significantly abolished the synergistic therapeutic effect. We hypothesize that following the concurrent activation of STAT1 by the two drugs, STAT1 functions as a transcription factor to further regulate the expression of related genes, thereby suppressing tumor growth. Additionally, we noted several reports indicating that EZH2 and HDAC can form complexes to exert direct effects, such as the EGR1/EZH2/HDAC9 complex and the EZH2/HDAC1 complex [50, 51]. These findings together support the practicality of combining EZH2 inhibitors with HDAC inhibitors.

In summary, our research elucidates that the use of EZH2 inhibitors may enhance the efficiency of HDAC inhibitors by diminishing H3K27me3 levels caused by HDAC inhibition. We also clarify potential mechanisms that operate for both EZH2 monotherapy and combination therapy in TCL, revealing the activation of STAT1 as one of the tumor-suppressive pathways contributing to the anti-tumor effects. Future study ought to attempt to characterize the molecular mechanisms of STAT1 in tumor cells, as well as its role in the tumor microenvironment. Additionally, it is crucial to thoroughly investigate the clinical value of combining EZH2 inhibitors with HDAC inhibitors or different therapy regimens. Our work establishes a foundation for such investigations and highlights the promise of targeted therapies in improving clinical outcomes for patients with T-cell lymphoma.

## Resource availability

### Materials availability

This study did not generate any unique reagents, all unique reagents generated in this study are available from the lead contact without restriction. This study did not generate any new original code.

## Supplementary information

Supplementary figures and figure legends

Supplementary material and methods

Western blots

Reproducibility checklist

## Data Availability

Sequencing data have been included in the supplementary information. This study did not generate any new original code. Additional information required to reanalyze the data reported in this paper can be obtained from the lead contact upon request.

## Code availability

Sequencing data have been included in the supplementary information. This study did not generate any new original code. Additional information required to reanalyze the data reported in this paper can be obtained from the lead contact upon request.
