# Supplementary material for: EZH2 inhibitor SHR2554 enhances the anti-tumor efficacy of HDAC inhibitor Chidamide through STAT1 in T-cell lymphoma
Source: Cell Death Dis. 2025 Jul 14;16(1):522. doi: 10.1038/s41419-025-07775-x (PMC12259945; doi:10.1038/s41419-025-07775-x)
Supplement: Supplementary file 1 — Supplementary figures and figure legends [file 41419_2025_7775_MOESM1_ESM.pdf]

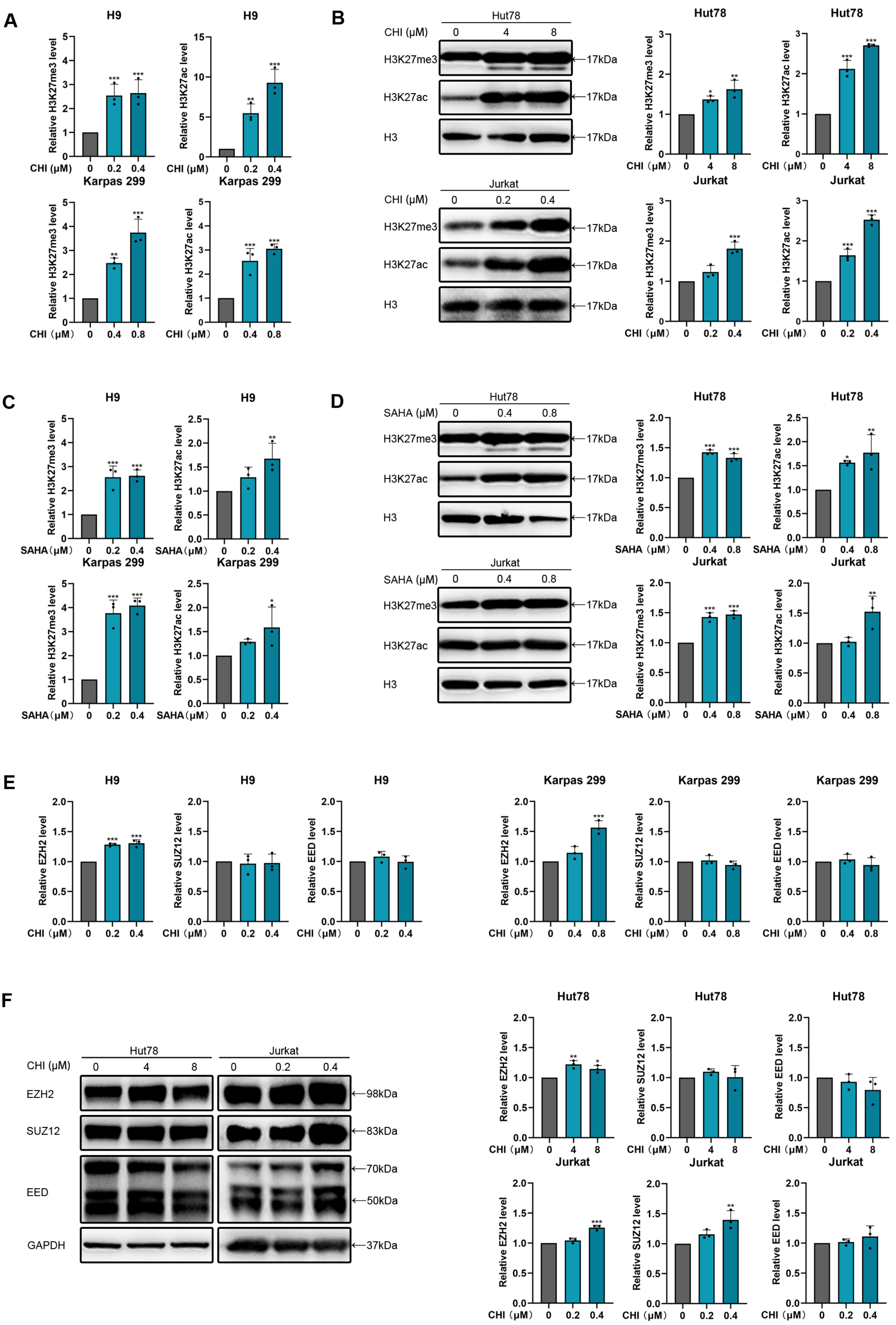

A
H9
Relative H3K27me3 level
Relative H3K27ac level
CHI (μM)
0
0.2
0.4
Karpas 299
0.8
B
Hut78
Jurkat
CHI (μM)
H3K27me3
H3K27ac
H3
17kDa
4
8
C
SAHA (μM)
D
E
Relative EZH2 level
Relative SUZ12 level
Relative EED level
F
EZH2
SUZ12
EED
GAPDH
98kDa
83kDa
70kDa
50kDa
37kDa

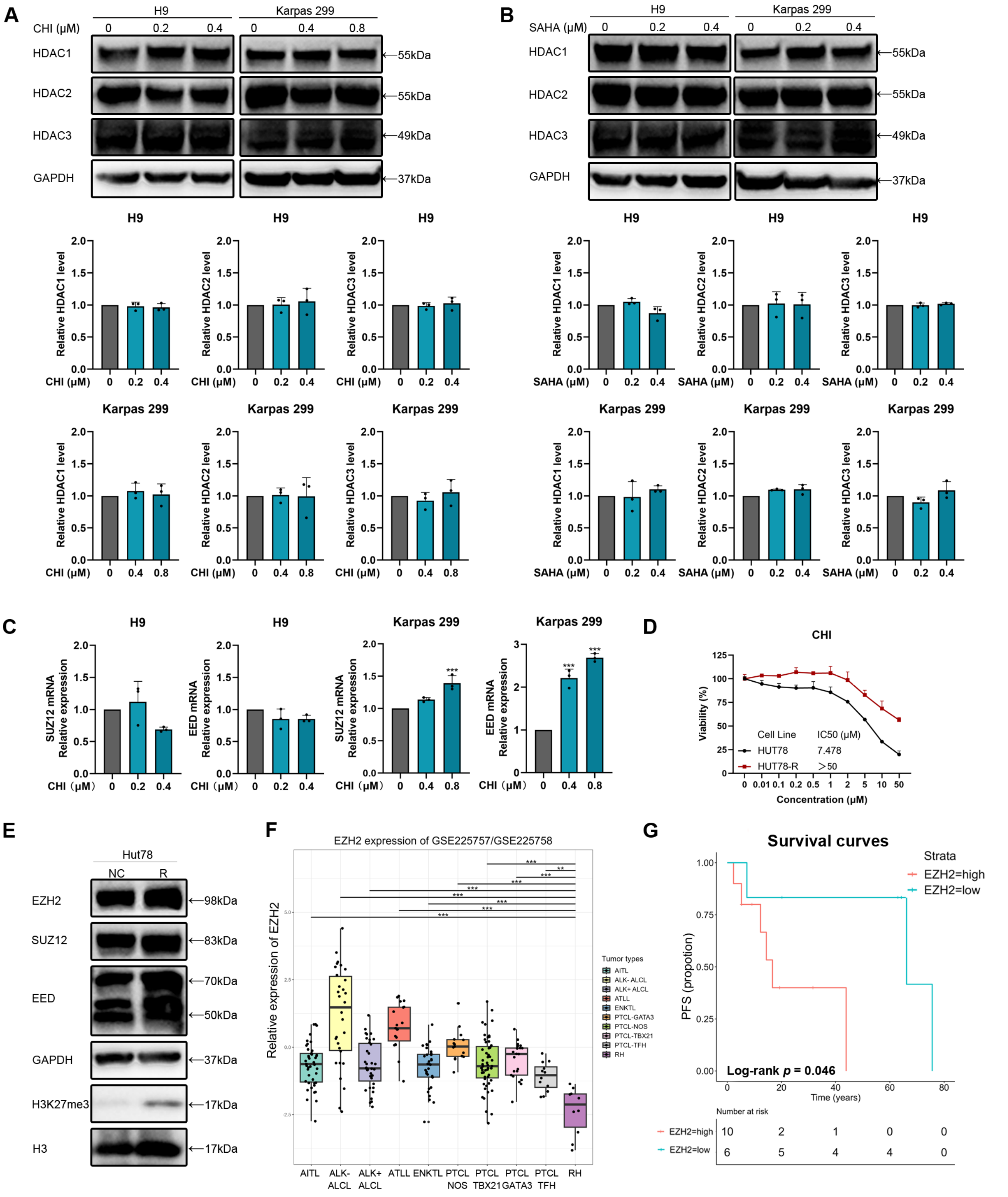
A
H9
Karpas 299
CHI (μM)
0 0.2 0.4
0 0.4 0.8
HDAC1
HDAC2
HDAC3
GAPDH
55kDa
55kDa
49kDa
37kDa
B
SAHA (μM)
0 0.2 0.4
0 0.2 0.4
Relative HDAC1 level
Relative HDAC2 level
Relative HDAC3 level
C
SUZ12 mRNA Relative expression
EED mRNA Relative expression
***
D
CHI
Viability (%)
Cell Line
IC50 (μM)
HUT78
7.478
HUT78-R
＞50
Concentration (μM)
0 0.01 0.1 0.2 0.5 1 2 5 10 50
E
Hut78
NC
R
EZH2
SUZ12
EED
GAPDH
H3K27me3
H3
98kDa
83kDa
70kDa
50kDa
37kDa
17kDa
17kDa
F
EZH2 expression of GSE225757/GSE225758
Relative expression of EZH2
Tumor types
AITL
ALK- ALCL
ALK+ ALCL
ATLL
ENKTL
PTCL-GATA3
PTCL-NOS
PTCL-TBX21
PTCL-TFH
RH
AITL
ALK- ALCL
ALK+ ALCL
ATLL
ENKTL
PTCL NOS
PTCL TBX21
PTCL GATA3
PTCL TFH
RH
G
Survival curves
Strata
EZH2=high
EZH2=low
PFS (propotion)
Log-rank p = 0.046
Time (years)
Number at risk
EZH2=high 10 2 1 0 0
EZH2=low 6 5 4 4 0

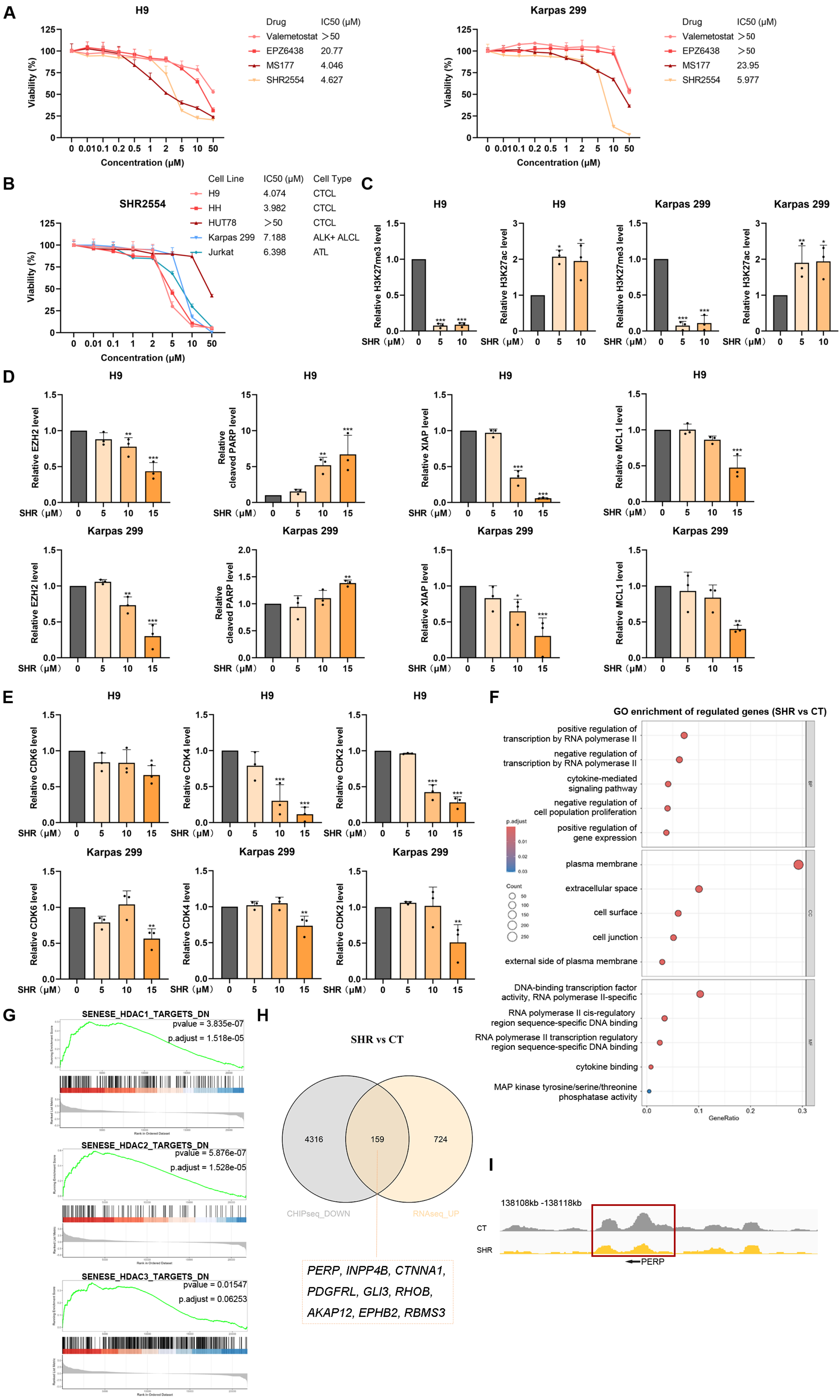

A
H9
Drug IC50 (μM)
Valemetostat >50
EPZ6438 20.77
MS177 4.046
SHR2554 4.627
Karpas 299
Drug IC50 (μM)
Valemetostat >50
EPZ6438 >50
MS177 23.95
SHR2554 5.977
Viability (%)
Concentration (μM)
B
SHR2554
Cell Line IC50 (μM) Cell Type
H9 4.074 CTCL
HH 3.982 CTCL
HUT78 >50 CTCL
Karpas 299 7.188 ALK+ ALCL
Jurkat 6.398 ATL
Viability (%)
Concentration (μM)
C
H9
Karpas 299
Relative H3K27me3 level
Relative H3K27ac level
SHR (μM)
D
H9
Karpas 299
Relative EZH2 level
Relative cleaved PARP level
Relative XIAP level
Relative MCL1 level
SHR (μM)
E
H9
Karpas 299
Relative CDK6 level
Relative CDK4 level
Relative CDK2 level
SHR (μM)
F
GO enrichment of regulated genes (SHR vs CT)
positive regulation of transcription by RNA polymerase II
negative regulation of transcription by RNA polymerase II
cytokine-mediated signaling pathway
negative regulation of cell population proliferation
positive regulation of gene expression
plasma membrane
extracellular space
cell surface
cell junction
external side of plasma membrane
DNA-binding transcription factor activity, RNA polymerase II-specific
RNA polymerase II cis-regulatory region sequence-specific DNA binding
RNA polymerase II transcription regulatory region sequence-specific DNA binding
cytokine binding
MAP kinase tyrosine/serine/threonine phosphatase activity
p.adjust
Count
GeneRatio
G
SENESE_HDAC1_TARGETS_DN
pvalue = 3.835e-07
p.adjust = 1.518e-05
SENESE_HDAC2_TARGETS_DN
pvalue = 5.876e-07
p.adjust = 1.528e-05
SENESE_HDAC3_TARGETS_DN
pvalue = 0.01547
p.adjust = 0.06253
H
SHR vs CT
4316
159
724
CHIPseq_DOWN
RNAseq_UP
PERP, INPP4B, CTNNA1, PDGFRL, GLI3, RHOB, AKAP12, EPHB2, RBMS3
I
138108kb -138118kb
CT
SHR
PERP

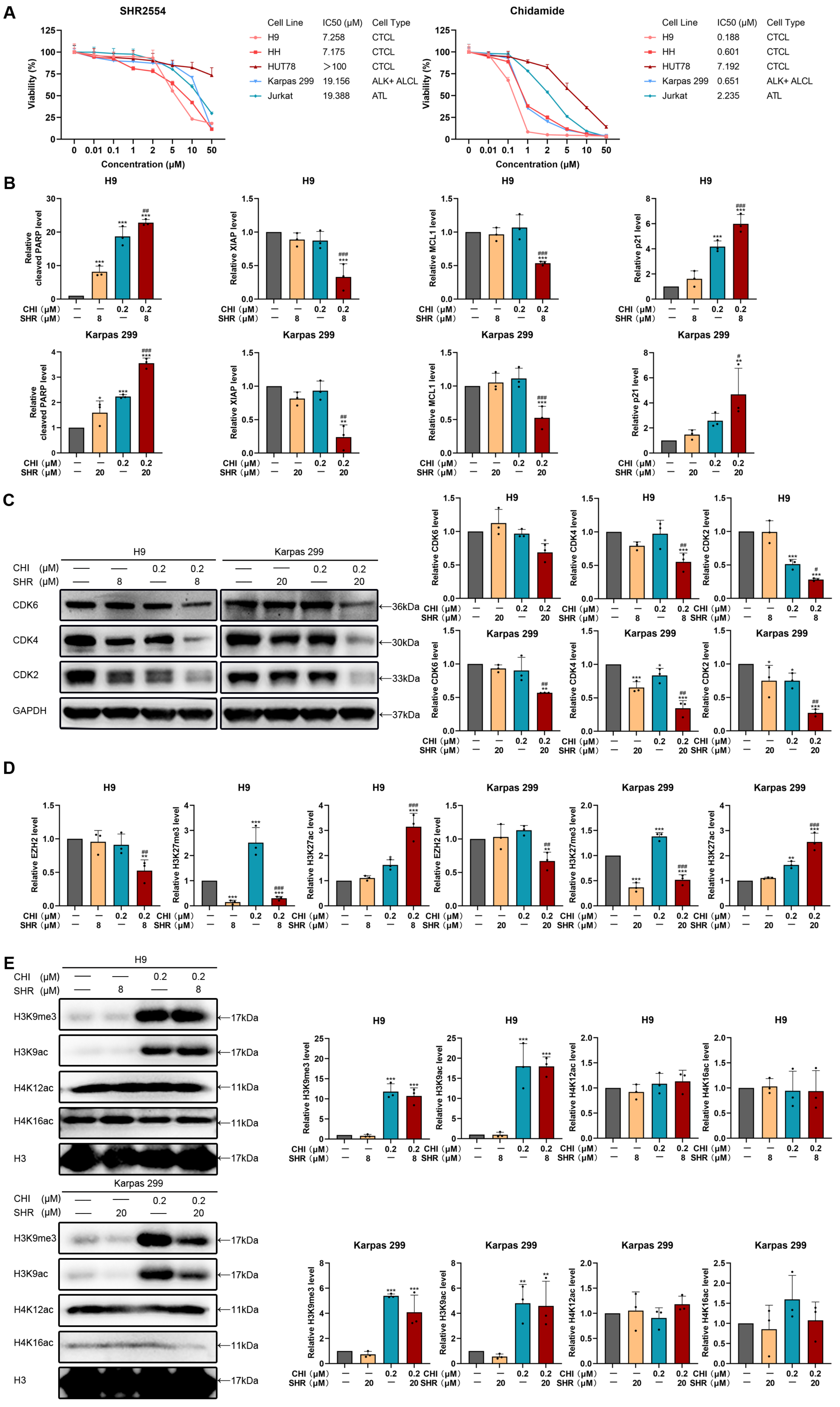
A
SHR2554
Chidamide
Viability (%)
Concentration (μM)
Cell Line IC50 (μM) Cell Type
H9 7.258 CTCL
HH 7.175 CTCL
HUT78 >100 CTCL
Karpas 299 19.156 ALK+ ALCL
Jurkat 19.388 ATL
Cell Line IC50 (μM) Cell Type
H9 0.188 CTCL
HH 0.601 CTCL
HUT78 7.192 CTCL
Karpas 299 0.651 ALK+ ALCL
Jurkat 2.235 ATL
B
H9
Karpas 299
Relative cleaved PARP level
Relative XIAP level
Relative MCL1 level
Relative p21 level
CHI (μM)
SHR (μM)
C
CDK6
CDK4
CDK2
GAPDH
36kDa
30kDa
33kDa
37kDa
Relative CDK6 level
Relative CDK4 level
Relative CDK2 level
D
Relative EZH2 level
Relative H3K27me3 level
Relative H3K27ac level
E
H3K9me3
H3K9ac
H4K12ac
H4K16ac
H3
17kDa
11kDa
Relative H3K9me3 level
Relative H3K9ac level
Relative H4K12ac level
Relative H4K16ac level

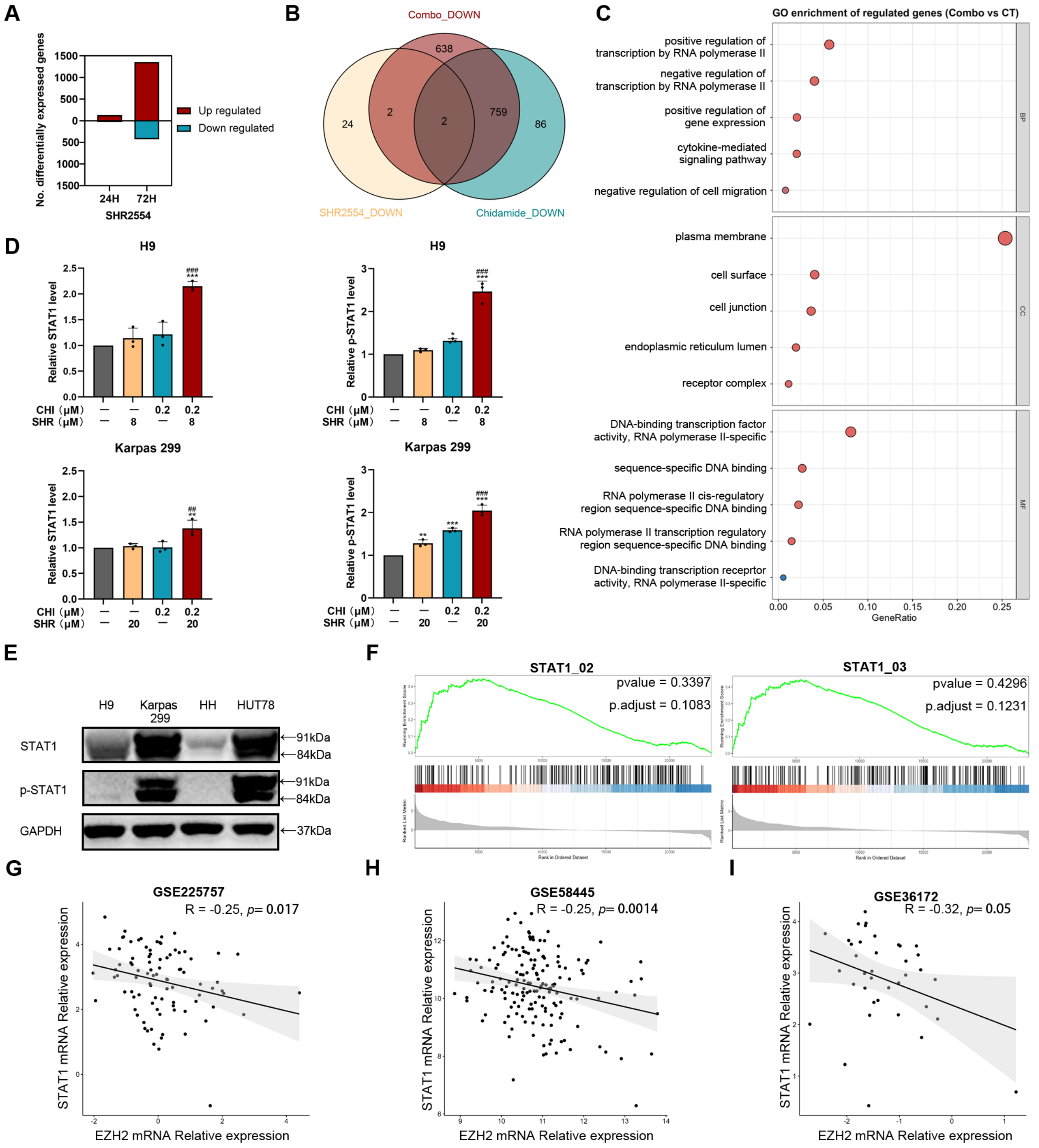

A
No. differentially expressed genes
1500
1000
500
0
500
1000
1500
24H
72H
SHR2554
Up regulated
Down regulated
B
Combo_DOWN
638
2
2
759
24
86
SHR2554_DOWN
Chidamide_DOWN
C
GO enrichment of regulated genes (Combo vs CT)
positive regulation of transcription by RNA polymerase II
negative regulation of transcription by RNA polymerase II
positive regulation of gene expression
cytokine-mediated signaling pathway
negative regulation of cell migration
plasma membrane
cell surface
cell junction
endoplasmic reticulum lumen
receptor complex
DNA-binding transcription factor activity, RNA polymerase II-specific
sequence-specific DNA binding
RNA polymerase II cis-regulatory region sequence-specific DNA binding
RNA polymerase II transcription regulatory region sequence-specific DNA binding
DNA-binding transcription receprtor activity, RNA polymerase II-specific
BP
CC
MF
0.00
0.05
0.10
0.15
0.20
0.25
GeneRatio
p.adjust
0.005
0.010
0.015
0.020
Count
300
600
900
D
H9
Relative STAT1 level
Relative p-STAT1 level
CHI (μM)
SHR (μM)
0.2
8
Karpas 299
20
E
H9
Karpas 299
HH
HUT78
STAT1
p-STAT1
GAPDH
91kDa
84kDa
37kDa
F
STAT1_02
pvalue = 0.3397
p.adjust = 0.1083
STAT1_03
pvalue = 0.4296
p.adjust = 0.1231
G
GSE225757
R = -0.25, p= 0.017
STAT1 mRNA Relative expression
EZH2 mRNA Relative expression
H
GSE58445
R = -0.25, p= 0.0014
I
GSE36172
R = -0.32, p= 0.05

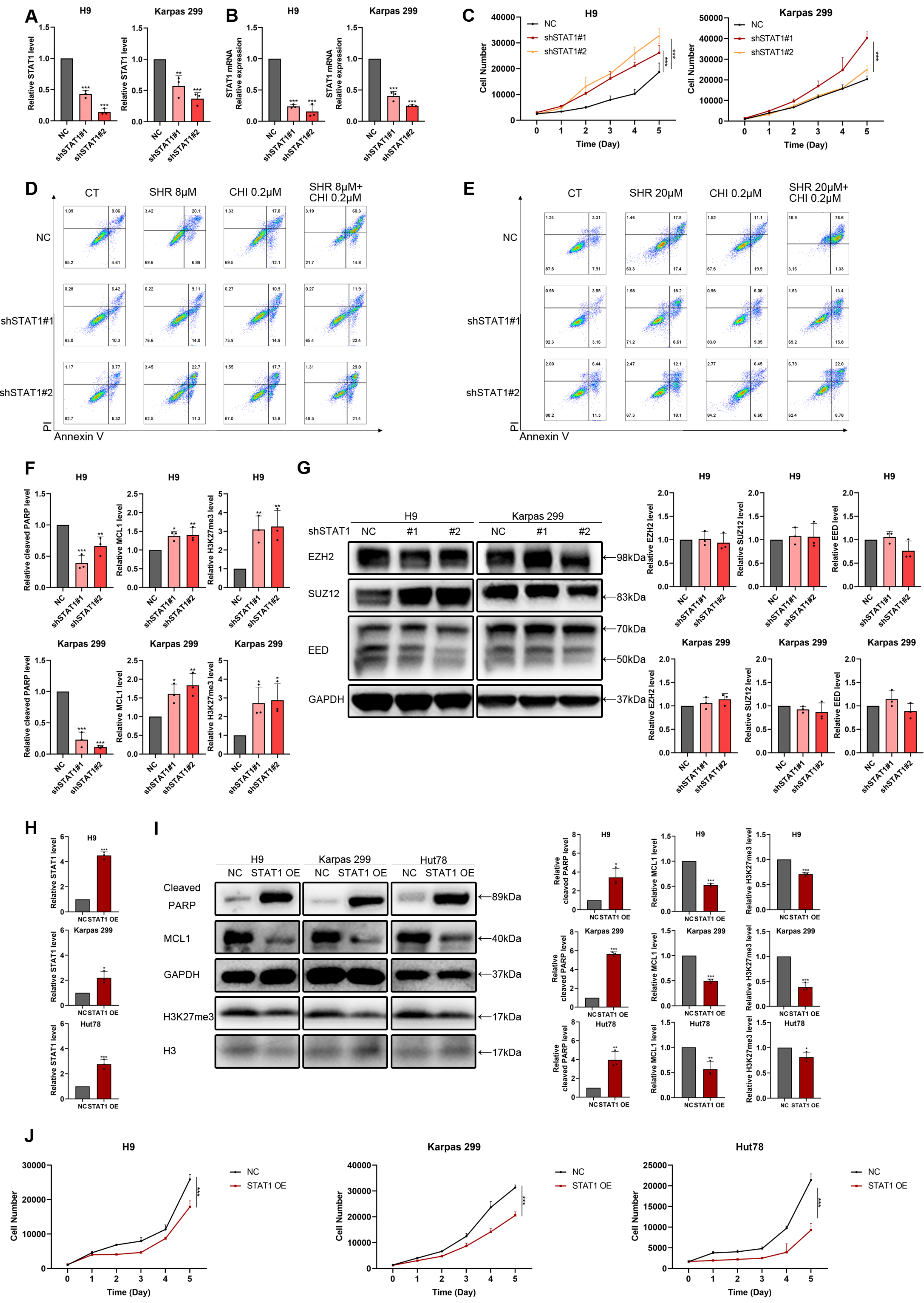
A
H9
Karpas 299
Relative STAT1 level
NC
shSTAT1#1
shSTAT1#2
B
STAT1 mRNA Relative expression
C
Cell Number
Time (Day)
D
CT
SHR 8μM
CHI 0.2μM
SHR 8μM+ CHI 0.2μM
PI
Annexin V
E
SHR 20μM
SHR 20μM+ CHI 0.2μM
F
Relative cleaved PARP level
Relative MCL1 level
Relative H3K27me3 level
G
shSTAT1
NC
#1
#2
EZH2
SUZ12
EED
GAPDH
98kDa
83kDa
70kDa
50kDa
37kDa
Relative EZH2 level
Relative SUZ12 level
Relative EED level
H
Hut78
STAT1 OE
I
Cleaved PARP
MCL1
H3K27me3
H3
89kDa
40kDa
17kDa
Relative cleaved PARP level
J

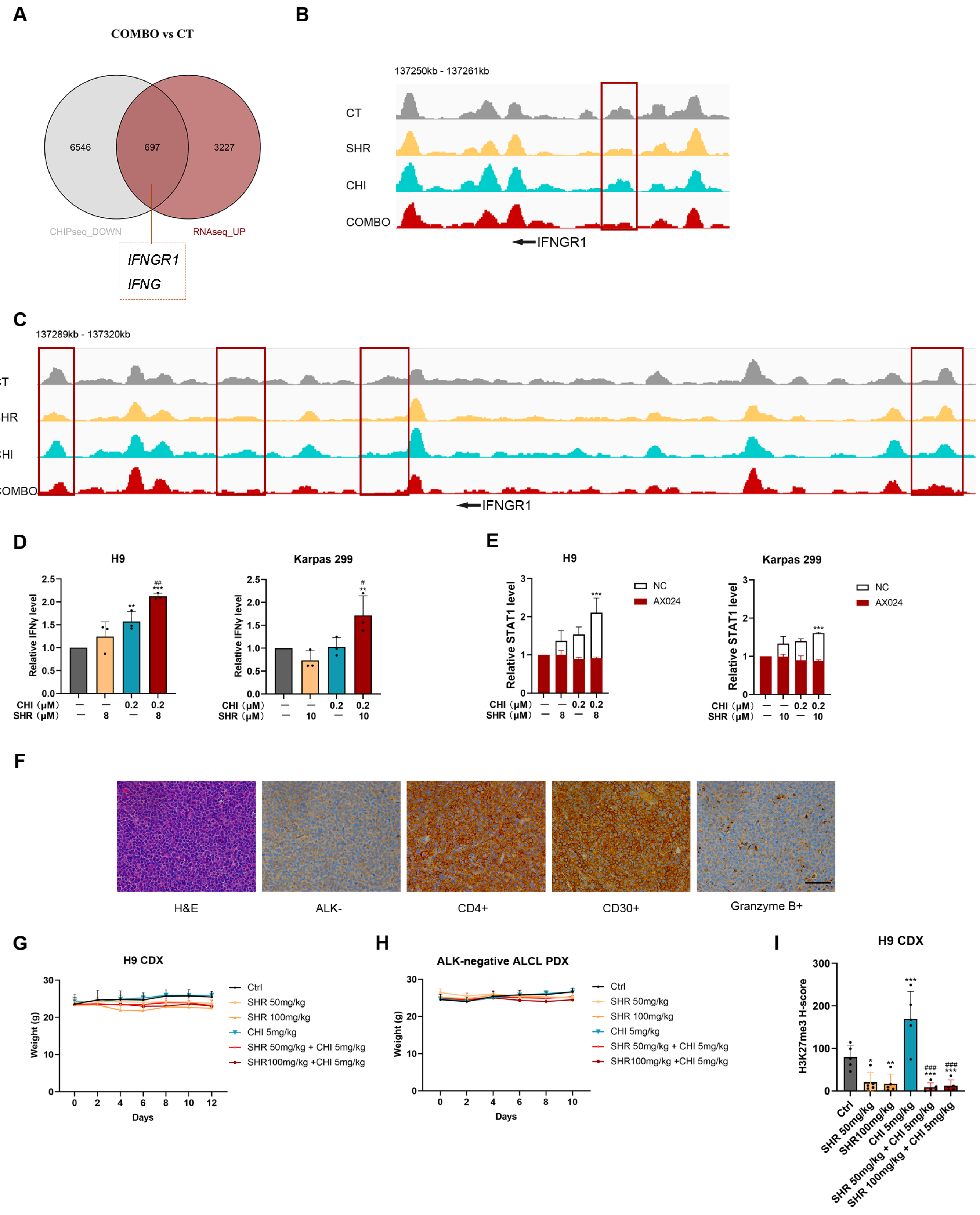
A
COMBO vs CT
6546
697
3227
CHIPseq_DOWN
RNAseq_UP
IFNGR1
IFNG
B
137250kb - 137261kb
CT
SHR
CHI
COMBO
IFNGR1
C
137289kb - 137320kb
CT
SHR
CHI
COMBO
IFNGR1
D
H9
Karpas 299
Relative IFNγ level
CHI（μM）
SHR（μM）
E
H9
Karpas 299
Relative STAT1 level
NC
AX024
CHI（μM）
SHR（μM）
F
H&E
ALK-
CD4+
CD30+
Granzyme B+
G
H9 CDX
Weight (g)
Days
Ctrl
SHR 50mg/kg
SHR 100mg/kg
CHI 5mg/kg
SHR 50mg/kg + CHI 5mg/kg
SHR100mg/kg +CHI 5mg/kg
H
ALK-negative ALCL PDX
Weight (g)
Days
Ctrl
SHR 50mg/kg
SHR 100mg/kg
CHI 5mg/kg
SHR 50mg/kg + CHI 5mg/kg
SHR100mg/kg +CHI 5mg/kg
I
H9 CDX
H3K27me3 H-score
Ctrl
SHR 50mg/kg
SHR100mg/kg
CHI 5mg/kg
SHR 50mg/kg + CHI 5mg/kg
SHR 100mg/kg + CHI 5mg/kg

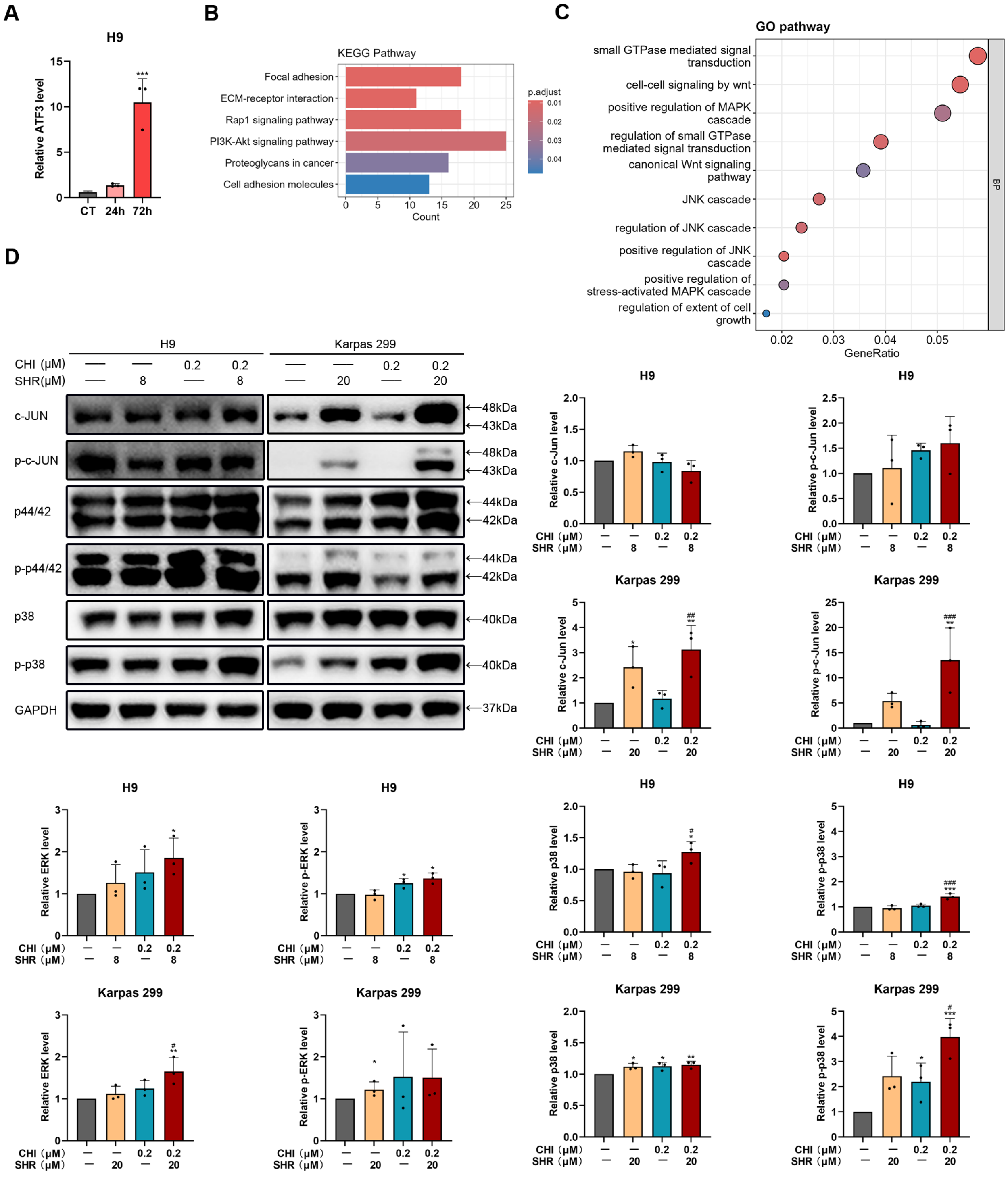
A
H9
Relative ATF3 level
CT
24h
72h
***
B
KEGG Pathway
Focal adhesion
ECM-receptor interaction
Rap1 signaling pathway
PI3K-Akt signaling pathway
Proteoglycans in cancer
Cell adhesion molecules
Count
p.adjust
C
GO pathway
small GTPase mediated signal transduction
cell-cell signaling by wnt
positive regulation of MAPK cascade
regulation of small GTPase mediated signal transduction
canonical Wnt signaling pathway
JNK cascade
regulation of JNK cascade
positive regulation of JNK cascade
positive regulation of stress-activated MAPK cascade
regulation of extent of cell growth
GeneRatio
BP
p.adjust
Count
D
H9
Karpas 299
CHI (μM)
SHR(μM)
c-JUN
p-c-JUN
p44/42
p-p44/42
p38
p-p38
GAPDH
48kDa
43kDa
44kDa
42kDa
40kDa
37kDa
Relative c-Jun level
Relative p-c-Jun level
Relative ERK level
Relative p-ERK level
Relative p38 level
Relative p-p38 level
CHI (μM)
SHR (μM)

Supplementary Figure S1. TCL tumor cells with acquired resistance to HDAC inhibitors exhibited elevated H3K27me3

A and C. Quantitative analysis of the protein expression results presented in Figure 1A (A) and Figure 1B (C).

B and D. Western blot analysis showed increased levels of H3K27me3 and H3K27ac following treatment with HDAC inhibitors Chidamide (B) and SAHA (D) in the Hut78 and Jurkat cell lines at the indicated concentrations for 48 h (1×10^5 cells/mL), compared to the DMSO control. Histone H3 (H3) was used as a loading control.

E. Quantitative analysis of the protein expression results presented in Figure 1C.

F. Western blot analysis was performed to assess the protein levels of the core subunits of the PRC2 complex in the Hut78 and Jurkat cell lines, including EZH2, SUZ12, and EED, following treatment with CHI. GAPDH was used as a loading control.

Supplementary Figure S2. EZH2 may play a crucial role in the progression of TCL

A and B. Western blot analysis was performed to assess the protein levels of HDAC1, HDAC2, and HDAC3 in the H9 and Karpas 299 cell lines following treatment with CHI (A) and SAHA (B). GAPDH was used as a loading control.

C. RT-PCR was performed to assess mRNA expression of SUZ12 and EED following treatment with CHI. GAPDH was used as a loading control.

D. Dose-response curves for CHI were generated, comparing drug-resistant Hut78 cell lines (R) with parental Hut78 cell lines.

E. Western blot analysis was performed to assess the variations in protein levels of EZH2, SUZ12,

EED, and H3K27me3 between drug-resistant Hut78 cell lines (R) and Hut78 cell lines (NC). GAPDH or H3 was used as a loading control.

F. Gene expression profiling analysis from GSE225757 and GSE225758 demonstrated significantly elevated mRNA levels of EZH2 in patients with TCL compared to those with reactive hyperplasia of lymph nodes (RH).

G. Kaplan–Meier curves for progression-free survival (PFS) of TCL patients based on EZH2 mRNA expression from Peking University Cancer Hospital. A log-rank test was performed to assess statistical differences between the two groups. Tick marks indicate censored data.

Supplementary Figure S3. The EZH2 inhibitor SHR2554 mediates therapeutic effects in TCL by reducing H3K27me3 levels

A. Dose-response curves for various EZH2 inhibitors after 72 h of treatment in H9 and Karpas 299 cell lines. Data are presented as mean ± SD. Valemetostat, EZH1/2 inhibitor. MS177, EZH2 PROTAC degrader. EPZ6438, EZH2 inhibitor. SHR2554, EZH2 inhibitor.

B. Dose-response curves for SHR after 72 h in five TCL cell lines.

C. Quantitative analysis of the protein expression results presented in Figure 2B.

D. Quantitative analysis of the protein expression results presented in Figure 2E.

E. Quantitative analysis of the protein expression results presented in Figure 2F.

F. GO enrichment analysis of regulated genes in the SHR group compared to the control group.

G. GSEA plot of SENESE_HDAC1_TARGETS_DN, SENESE_HDAC2_TARGETS_DN, and SENESE_HDAC3_TARGETS_DN gene sets of comparison between SHR and the control group.

H. Venn diagram illustrating the downregulated genes of H3K27me3 from ChIP-seq and the

upregulated genes from RNA-seq in the SHR group compared to the control group.

I. IGV profiles demonstrating downregulated H3K27me3 occupancy at the PERP gene in the SHR group compared to the control group.

Supplementary Figure S4. SHR2554 enhances the efficacy of Chidamide by neutralizing HDAC inhibitor-induced elevations in H3K27me3 levels

A. Dose-response curves for SHR (left) and CHI (right) of 48 h in five TCL cell lines.

B. Quantitative analysis of the protein expression results presented in Figure 3C.

C. Western blot analysis was performed to assess cell cycle-related proteins. GAPDH was used as a loading control.

D. Quantitative analysis of the protein expression results presented in Figure 3E.

E. Western blot analysis was performed to assess histone modifications, including H3K9me3, H3K9ac, H4K12ac, and H4K16ac. H3 was used as a loading control.

Supplementary Figure S5. STAT1 upregulation occurred in response to the combination of SHR2554 and Chidamide

A. Histograms illustrating the changes in gene expression in the SHR group treated for 24 h and 72 h compared to the control group in RNA-seq analysis.

B. Venn diagram of the overlapping downregulated genes ($Log_2FC < -1$, $FDR < 0.05$) from different treatment groups in RNA-seq.

C. GO enrichment of regulated genes in the combo group compared to the control group from RNA-seq.

D. Quantitative analysis of the protein expression results presented in Figure 4C.

E. Western blot analysis was performed to validate the protein expression levels of STAT1 in different cell lines.

F. GSEA plot of STAT1_02 and STAT1_03 gene sets of comparison between combo and the control group.

G-I. The negative correlation between the relative mRNA expression of EZH2 and STAT1 in GSE225757 (G), GSE58445 (H), and GSE36172 (I) is presented.

Supplementary Figure S6. SHR2554 and Chidamide exert a synergistic effect through upregulation of STAT1

A. Quantitative analysis of the protein expression results presented in Figure 4E.

B. STAT1 knockdown via shRNA was validated using RT-PCR. GAPDH was used as a loading control.

C. The growth curve of tumor cells (initiated at 2×10^3 cells/well) with STAT1 knockdown achieved through shRNA lentivirus. Cell numbers were assessed using the CellTiter-Glo luminescent cell viability assay.

D and E. Flow cytometry analysis of the apoptosis ratio in H9 (D) and Karpas 299 (E) cell lines with STAT1 knockout.

F. Quantitative analysis of the protein expression results presented in Figure 4H.

G. Western blot analysis was performed to assess variations in protein levels of EZH2, SUZ12, and EED in STAT1 knockdown cell lines. GAPDH was used as a loading control.

H. Quantitative analysis of the protein expression results presented in Figure 4I.

I. Western blot analysis was performed to assess apoptosis-related proteins and H3K27 modifications in STAT1 overexpression cell lines. GAPDH or H3 was used as a loading control.

J. The growth curve of tumor cells (initiated at 2×10^3 cells/well) with STAT1 overexpression.

Supplementary Figure S7. The loss of H3K27me3 at the IFNG locus enhances IFN-γ expression and activates STAT1

A. Venn diagram illustrating the downregulated genes of H3K27me3 from ChIP-seq and the upregulated genes from RNA-seq in the combo group compared to the control group.

B and C. IGV profiles demonstrating downregulated H3K27me3 occupancy at different IFNGR1 sites in each treatment group.

D. Quantitative analysis of the IFNγ protein expression results presented in Figure 4N.

E. Quantitative analysis of the STAT1 protein variations presented in Figure 4N.

F. Results of H&E staining and immunohistochemical staining for ALK, CD4, CD30, and granzyme B in ALK-negative ALCL PDX.

G and H. Changes of body weight in NCG mice during treatment.

I. Statistics data represent of H-score of H3K27me3 for each group.

Supplementary Figure S8. Aberrant activation of the MAPK pathway in the combination therapy

A. Histograms illustrating the changes in ATF3 gene expression in the SHR group treated for 24 h and 72 h compared to the control group in RNA-seq analysis.

B and C. KEGG (B) and GO (C) enrichment of genes in the overlapping region of Supplementary

Figure S7A.

D. Western blot was performed to validate abnormal activation of MAPK signaling pathway-related proteins in the combo group. GAPDH was used as a loading control.
