# Supplementary material for: EZH2 inhibitor SHR2554 enhances the anti-tumor efficacy of HDAC inhibitor Chidamide through STAT1 in T-cell lymphoma
Source: Cell Death Dis. 2025 Jul 14;16(1):522. doi: 10.1038/s41419-025-07775-x (PMC12259945; doi:10.1038/s41419-025-07775-x)
Supplement: Supplementary file 2 — Supplementary material and methods [file 41419_2025_7775_MOESM2_ESM.pdf]

Methods and Materials

Cell lines and reagents

The human T-cell lymphoma cell lines Jurkat, Hut78, and Karpas 299 used in this study were generously provided by Dr. Fu Kai (MD Anderson Cancer Center, USA). The H9 and HH cell lines were purchased from the American Type Culture Collection (Virginia, USA). HEK293T cells were maintained in our laboratory. All cell lines were authenticated by STR profiling and confirmed to be free of mycoplasma contamination. RPMI-1640, IMDM, and DMEM high glucose media (Gibco, Thermo Fisher Scientific, Massachusetts, USA) were supplemented with 10% fetal bovine serum (Biological Industries, Sartorius, Israel, #C04001) and 0.5% penicillin/streptomycin (Biological Industries, #C3420) as the basic media for cell culture. All cell lines were cultured in a 5% CO_2 incubator at 37°C.

Chidamide (CS055/HBI-8000) was kindly supplied by Chipscreen Biosciences Ltd. (Shenzhen, China), while the EZH2 inhibitor SHR2554 was provided by Jiangsu Hengrui Medicine Co., Ltd. Jiangsu, China). Vorinostat (Selleck, S1047) and AX-024 (Selleck, S6727) were purchased from Selleck (Houston, USA). Valemetostat (MCE, HY-109108), MS177 (MCE, HY-148333), and EPZ6438 (MCE, HY-13803) were purchased from MedChemExpress (New Jersey, USA). All compounds were dissolved in dimethyl sulfoxide (DMSO) (Sigma-Aldrich, Darmstadt, Germany) at a concentration of 10 mM and stored at −80 °C. Immunohistochemistry (IHC) staining was performed using the following antibodies: anti-Ki-67 (CST, Massachusetts, USA, #9449, 1:1500), anti-H3K27me3 (CST, #9733, 1:200). For Western blot (WB) detection, antibodies against H3K27me3 (CST, #9733), H3K27ac (CST, #8173), H3 (CST, #4499), EZH2 (CST, #5246), SUZ12 (CST, #3737), EED (CST, #85322), GAPDH (CST, #2118), active forms of poly-ADP ribose

polymerase (Cleaved PARP, CST, #9532), X-linked inhibitor of apoptosis protein (XIAP, CST, #2045), MCL-1 (CST, #5453), CDK2 (CST, #2546), CDK4 (CST, #12790), CDK6 (CST, #13331), STAT1 (CST, #14994), phospho-STAT1 (Tyr701) (CST, #7649), c-JUN (CST, #9165), phospho-c-JUN (Ser73) (CST, #3270), p38 MAPK (CST, #8690), phospho-p38 MAPK (Thr180/Tyr182) (CST, #4511), p44/42 MAPK (CST, #4695), phospho-p44/42 MAPK (Thr202/Tyr204) (CST, #4370), HDAC1 (Proteintech, 10197-1-AP), HDAC2 (Proteintech, 12922-3-AP), HDAC3 (Proteintech, 10255-1-AP), IFN-γ (CST, #98139), H3K9me3 (CST, #13969), H3K9ac (CST, #9649), H4K12ac (CST, #13944), and H4K16ac (CST, #13534) were used.

Immunohistochemistry (IHC)

Formalin-fixed and paraffin-embedded tumor samples were sectioned into 4-5 μm-thick slices for immunohistochemical (IHC) staining. The slides were incubated overnight at 4°C with a specific primary antibody, followed by incubation with a horseradish peroxidase-conjugated secondary antibody at room temperature for 40 minutes the next day. Diaminobenzidine (DAB) was used for visualization. The staining intensity of patient samples was quantified using QuPath v0.5.1 (Queen's University Belfast, UK), a bioimage analysis software designed for quantifying staining intensity [1]. Based on the mean cytoplasmic DAB optical density (OD) and the reported method, we calculated the H-score (H-score = 0 * negative cells % + 1 * (1+) cells % + 2 * (2+) cells % + 3 * (3+) cells %) for further analysis [2].

RNA extraction, real-time PCR, and Western blot analysis

For real-time PCR (RT-PCR) and Western blot (WB) analysis, cells were treated with various

durations and concentrations of SHR2554 and Chidamide, followed by collection and lysis using appropriate reagents. For RNA extraction and RT-PCR, cells were lysed with TRIzol (Invitrogen, Thermo Scientific, Massachusetts, USA, #15596018), and subsequent processing adhered to standard protocols. Primers were synthesized by Oligo Chem (Beijing, China). For WB analysis, cells were lysed using RIPA buffer (Invitrogen, Thermo Scientific, #89900), supplemented with a protease and phosphatase inhibitor cocktail (Roche, Basel, Switzerland, #04693132001 and #04906837001). Protein expression levels were quantified using ImageJ software (NIH) following standardized protocols.

Lentivirus packing and infection

Lentiviral vectors (pLKO-Tet-On) encoding STAT1-specific short hairpin RNA (shSTAT1; #1: 5′-GCUGGAUGAUCAAUAUAGUTT-3′; #2: 5′-GUGGCAAAGAGUGAUCAGATT-3′) were constructed by the Public Protein/Plasmid Library (Jiangsu, China). The STAT1 overexpression plasmid was also designed and produced by the Public Protein/Plasmid Library. HEK293T cells were seeded at a density of 1×10^7 cells in per 15 cm dish. After 24 hours, the transfection plasmids were mixed in Opti-MEM (Gibco, Massachusetts, USA, #51985034) to micrograms of DNA complex. After 48 and 72 hours, the media containing lentivirus was harvested and concentrated. The concentrated virus was used to infect cells in the presence of 8 μg/mL polybrene, followed by selection with puromycin (LabLead, #P0671). Infection efficiency was assessed using RT-PCR and Western blot analysis.

RNA-seq

mRNA sequencing was conducted by Annoroad Gene Technology (Beijing, China) using the Illumina platform (HiSeq system, PE150 sequencing strategy). For RNA sequencing, the reference genome was constructed with Bowtie (version 1.0.1), and the cleaned data were aligned to the reference genome using HISAT2 (version 2.1.0). Differential gene expression analysis was performed using DEGSeq (version 1.18.0), with an adjusted p-value threshold of < 0.05 considered statistically significant. Gene Ontology (GO), Kyoto Encyclopedia of Genes and Genomes (KEGG), and Gene Set Enrichment Analysis (GSEA) were conducted using the R packages *clusterProfiler* and *enrichplot*.

ChIP-seq

The chromatin immunoprecipitation (ChIP) assay was conducted using the SimpleChIP Plus Enzymatic Chromatin IP Kit (CST, #9005) according to the manufacturer's protocols. Approximately 4×10^6 cells subjected to various treatments from a 15-cm culture dish were utilized for each assay. The cells were fixed with 1% formaldehyde at room temperature for 10 minutes. Fixation was subsequently quenched by the addition of 2 mL of 10× glycine, followed by an additional 5 minutes of incubation at room temperature. The cells were then scraped, lysed, digested, and sheared via sonication. Lysates were clarified by centrifugation, and 2% of the supernatant was reserved as the input sample, which could be stored at –20 °C for future use. The supernatant was incubated overnight at +4 °C with immunoprecipitating antibodies, specifically antibodies against H3K27me3 (CST, #9733) and H3 (CST, #4499). Following this incubation, 30 μL of Protein G Magnetic Beads were added to each immunoprecipitation reaction, which was then rotated and incubated for 2 hours at 4 °C. After washing the beads, chromatin was eluted from the

antibody/protein G magnetic beads.

For all samples, including the 2% input sample obtained from previous steps, crosslinks were reversed by the addition of 6 μL of 5 M NaCl and 2 μL of Proteinase K, followed by a 2-hour incubation at 65 °C. DNA purification was performed using spin columns. The purity of the DNA was assessed using the NanoPhotometer® spectrophotometer (IMPLEN, CA, USA), while the DNA concentration was quantified using the Qubit® DNA Assay Kit in a Qubit® 3.0 Fluorometer (Life Technologies, CA, USA). The purified DNA was subsequently employed for ChIP-seq library preparation.

The library was constructed by Novogene Corporation (Beijing, China). Paired-end sequencing of the samples was then performed on the Illumina platform (Illumina, CA, USA). Library quality was evaluated using the Agilent Bioanalyzer 2100 system. Reference genome and gene model annotation files were directly downloaded from the genome website. The index for the reference genome was constructed using BWA (v 0.7.12), and the clean reads were aligned to the reference genome using BWA mem (v 0.7.12). For specific ChIP-seq binding sites, individual reads that mapped to either the plus or minus strand exhibited significant enrichment. In the case of single-end sequencing, fragment sizes were estimated using the MACS2 (Zhang et al., 2008) predicted method with default parameters. After mapping the reads to the reference genome, the MACS2 (version 2.1.0) peak calling software was employed to identify regions of immunoprecipitation (IP) enrichment above background levels. A q-value threshold of 0.05 was applied to all datasets. Following peak calling, the distributions of chromosome localization, peak width, fold enrichment, significance levels, and peak summit counts per peak were all analyzed and displayed.

Statistical analysis

Data representative of the findings are presented in the figures, with all values expressed as mean ± SD. Statistical analyses were performed using SPSS software. The chi-square test was used for categorical variables, while the Student's t-test was applied to compare two independent continuous variables. Additionally, one-way ANOVA was applied to evaluate differences among multiple groups, followed by post hoc comparisons. Survival analysis for individual genes or proteins was conducted using the log rank test. Correlation analyses were performed using the Spearman test for categorical variables and the Pearson test for continuous variables. Statistical significance was assumed for $p < 0.05$. All experiments were repeated biologically three times.

References

1. Bankhead P, Loughrey MB, Fernández JA, Dombrowski Y, McArt DG, Dunne PD*, et al.* QuPath: Open source software for digital pathology image analysis. *Sci Rep* 2017; **7**(1)**:** 16878.

2. Perdrix Rosell A, Maiques O, Martin JAJ, Chakravarty P, Ombrato L, Sanz-Moreno V*, et al.* Early functional mismatch between breast cancer cells and their tumour microenvironment suppresses long term growth. *Cancer Lett* 2022; **544:** 215800.
