# Supplementary material for: EZH2 inhibitor SHR2554 enhances the anti-tumor efficacy of HDAC inhibitor Chidamide through STAT1 in T-cell lymphoma
Source: Cell Death Dis. 2025 Jul 14;16(1):522. doi: 10.1038/s41419-025-07775-x (PMC12259945; doi:10.1038/s41419-025-07775-x)
Supplement: Supplementary file 3 — Western blots [file 41419_2025_7775_MOESM3_ESM.pdf]

Fig. 1a-b

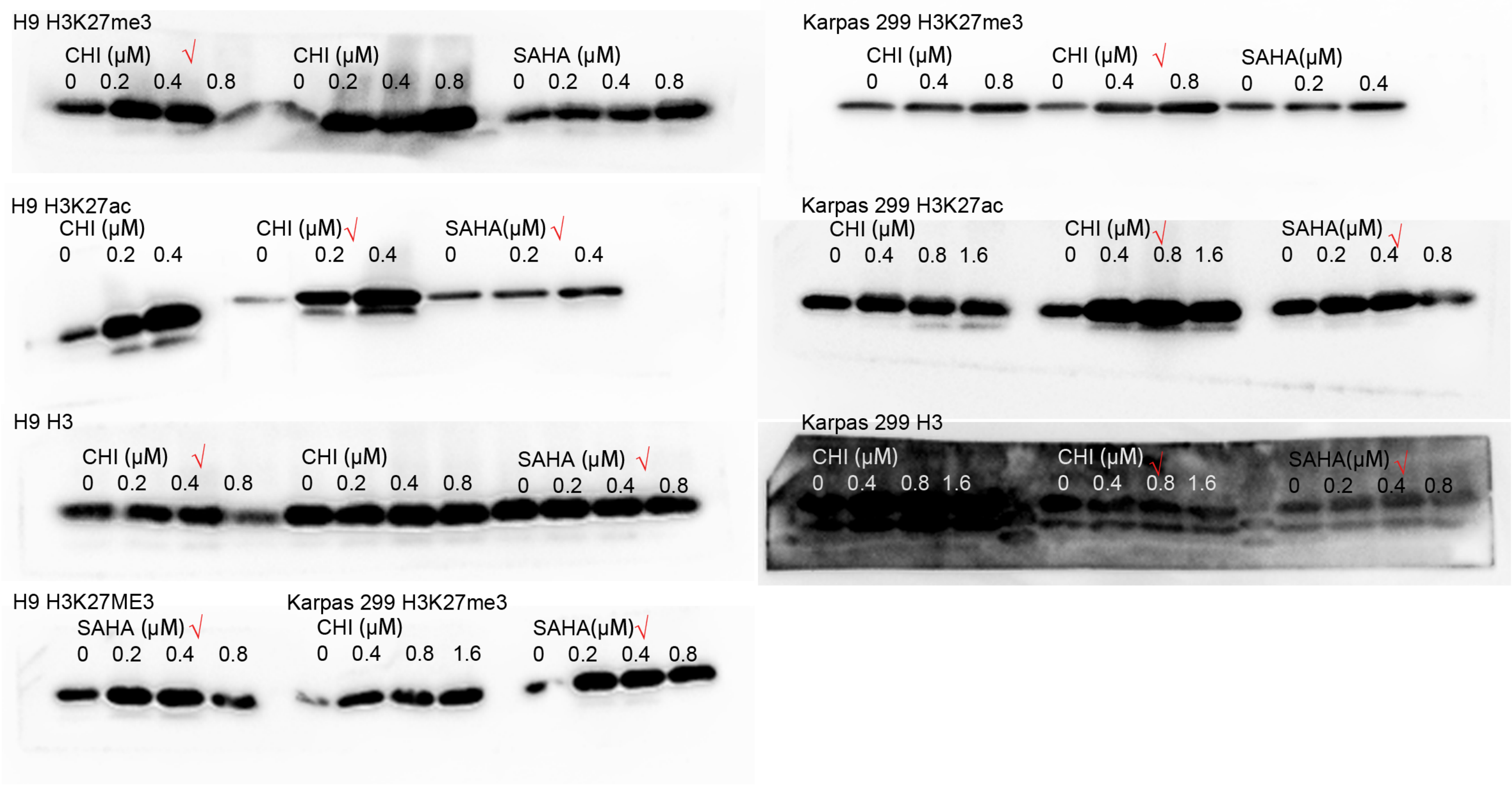

Fig. 1c

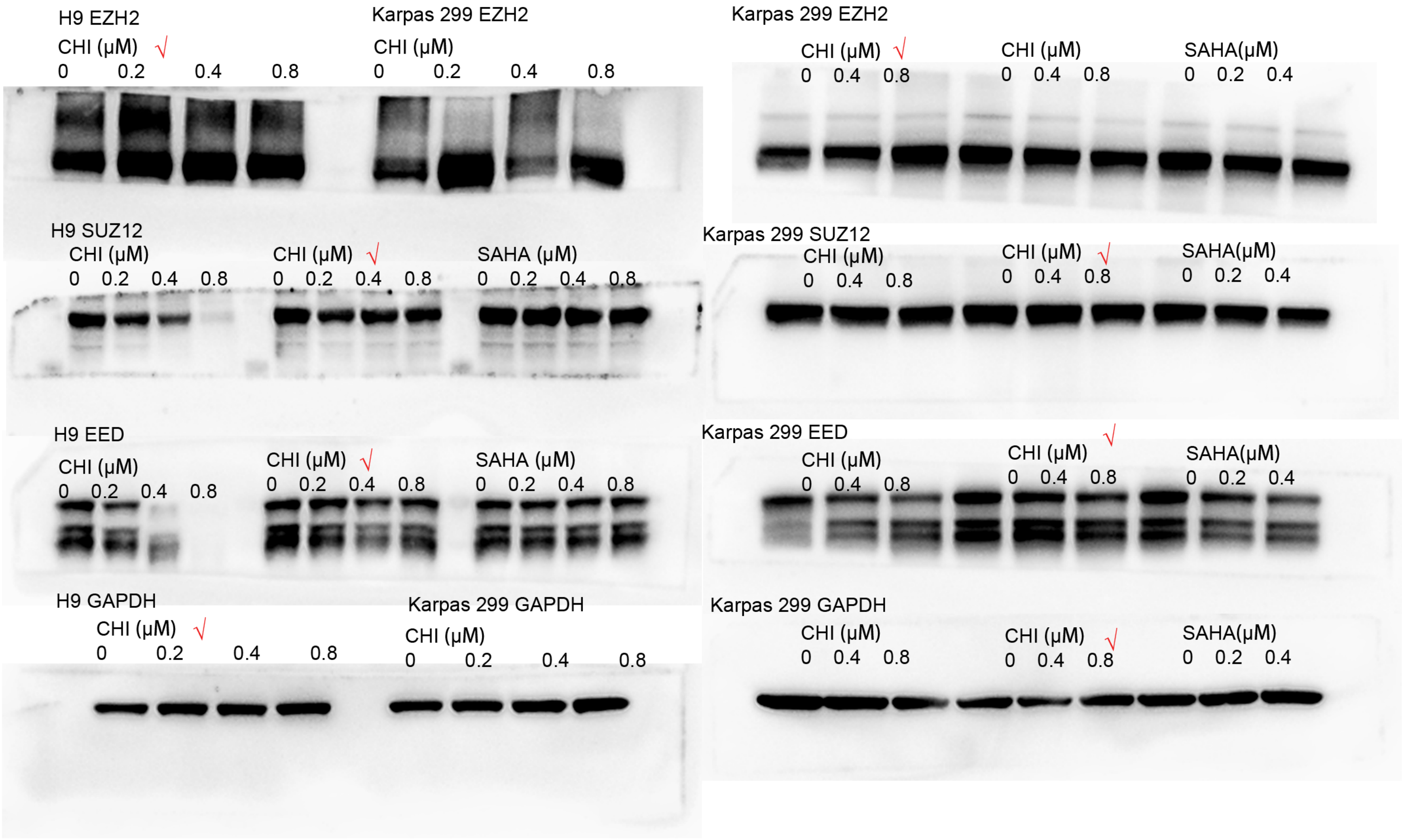

Fig. 1g, Fig. S2e

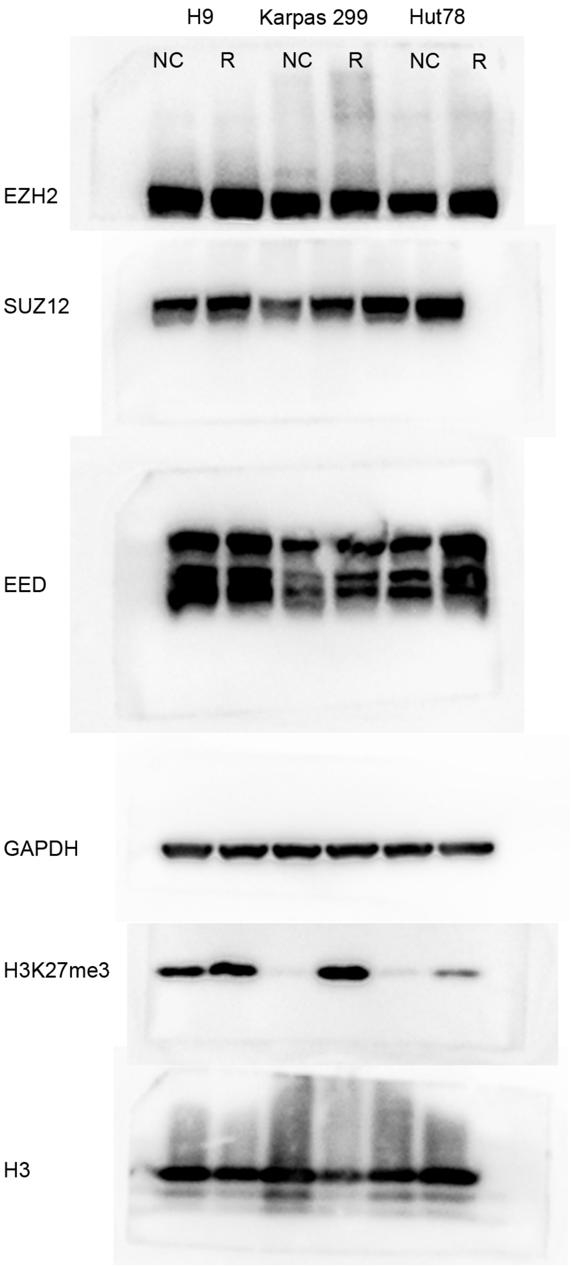

Fig. 1j

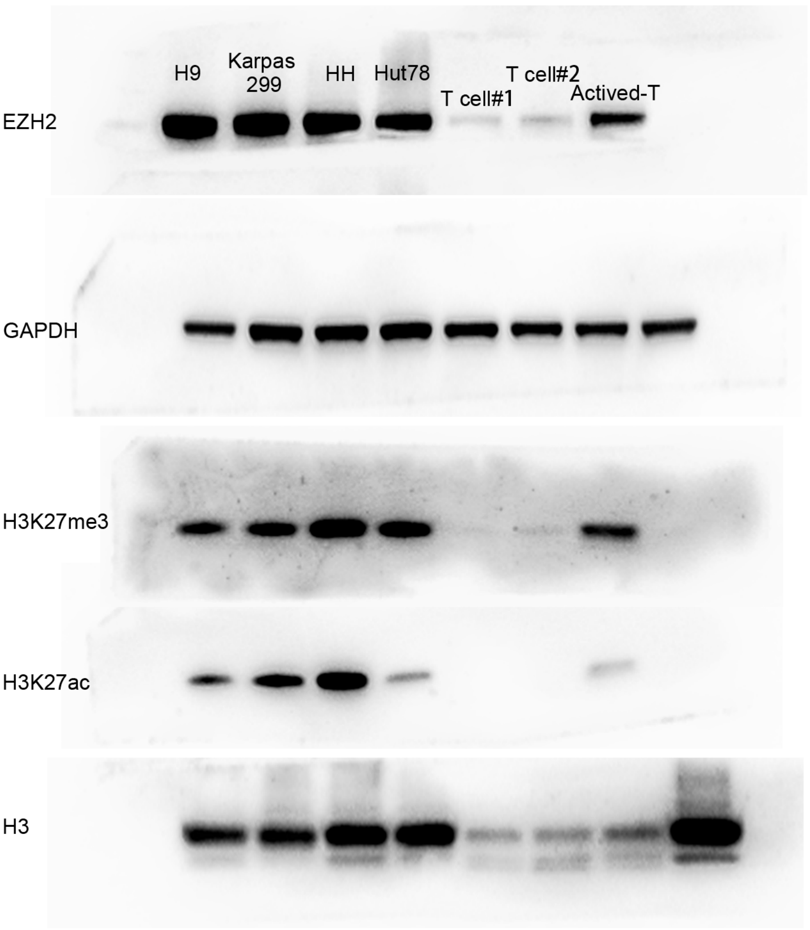

Fig. 2b

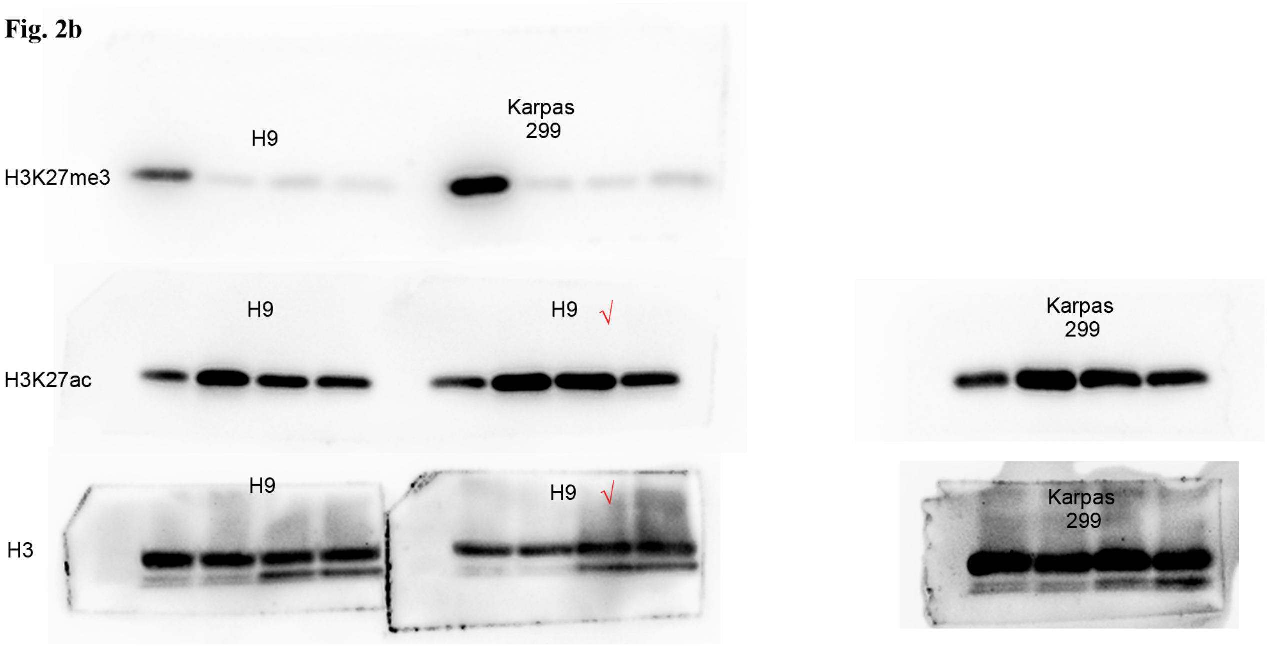
H9
Karpas 299
H3K27me3
H9
H9 √
Karpas 299
H3K27ac
H9
H9 √
Karpas 299
H3

Fig. 2e

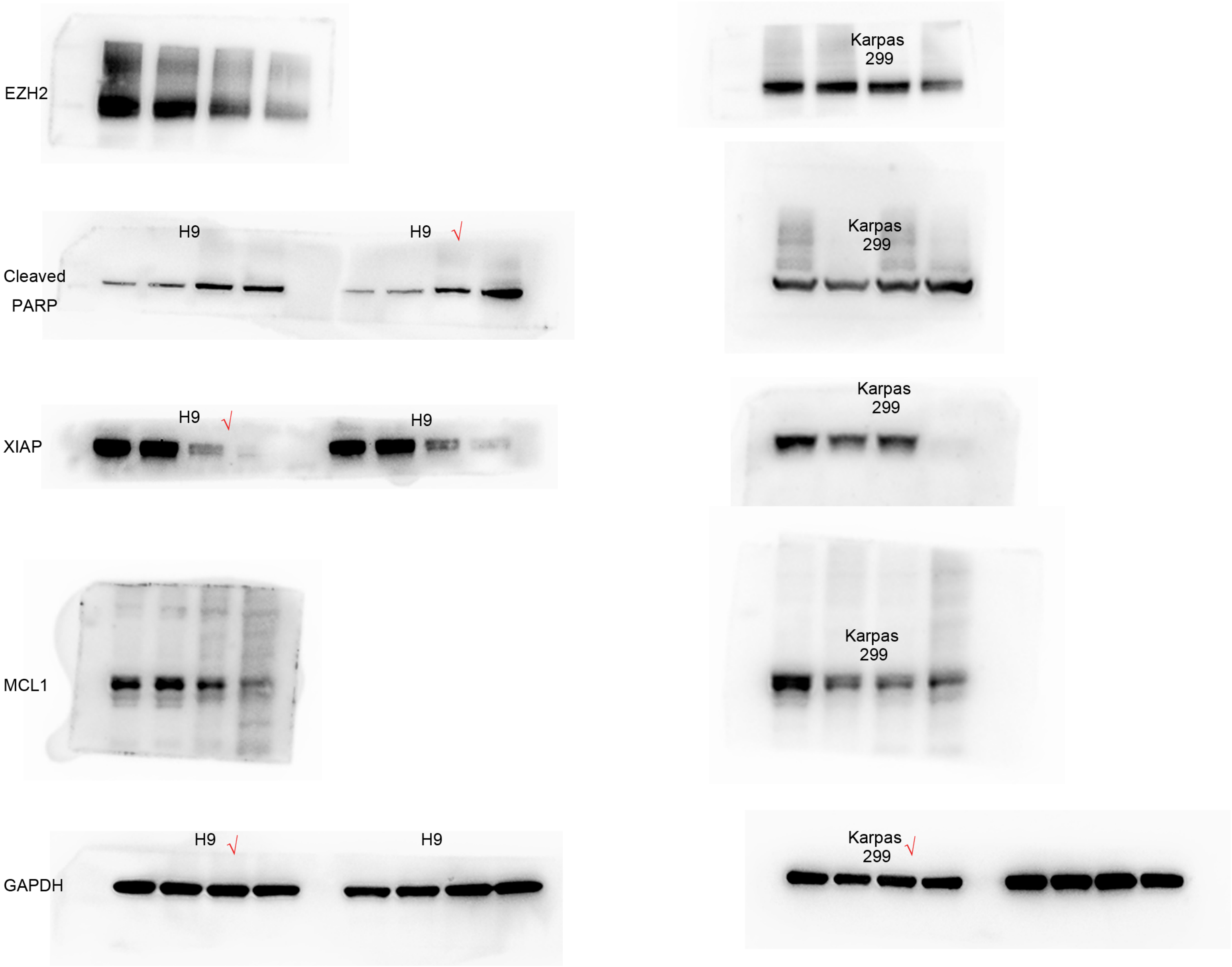
EZH2
Karpas 299
H9
H9 √
Karpas 299
Cleaved PARP
H9 √
H9
Karpas 299
XIAP
Karpas 299
MCL1
H9 √
H9
Karpas 299 √
GAPDH

Fig. 2f

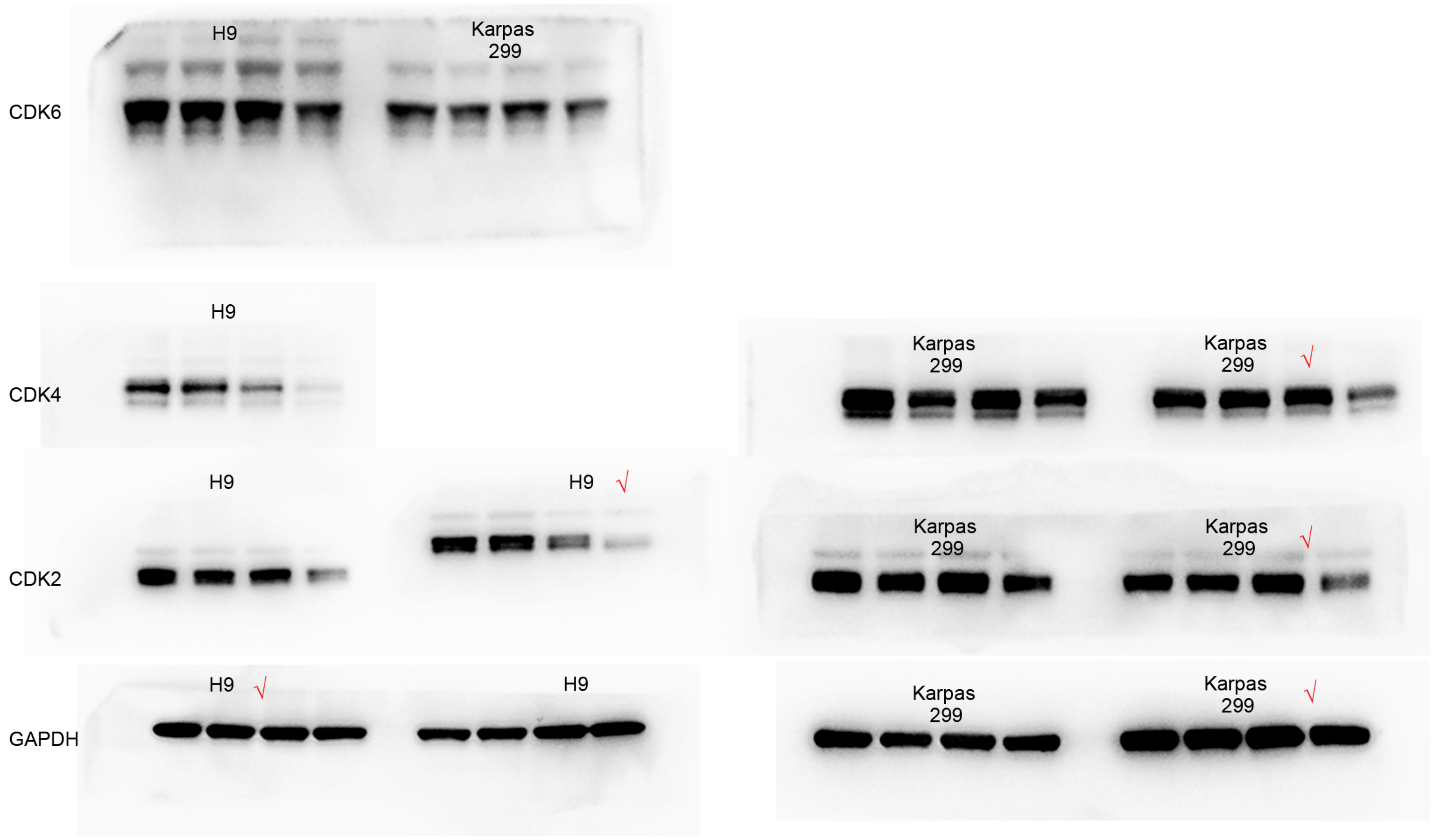

CDK6
H9
Karpas 299
CDK4
H9
Karpas 299
Karpas 299 √
CDK2
H9
H9 √
Karpas 299
Karpas 299 √
GAPDH
H9 √
H9
Karpas 299
Karpas 299 √

Fig. 3c

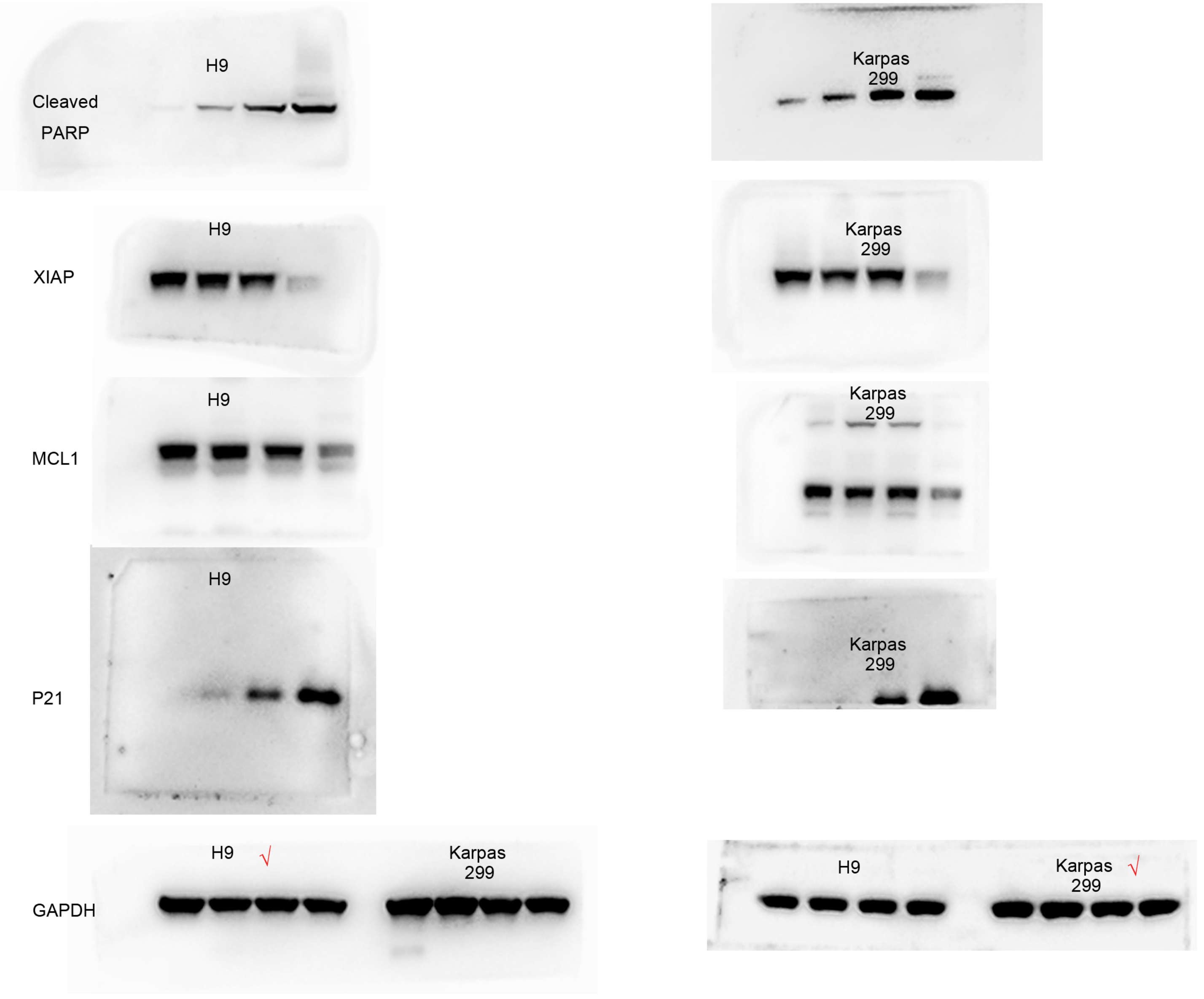

Cleaved PARP
H9
Karpas 299
XIAP
H9
Karpas 299
MCL1
H9
Karpas 299
P21
H9
Karpas 299
GAPDH
H9 √
Karpas 299
H9
Karpas 299 √

Fig. 3e

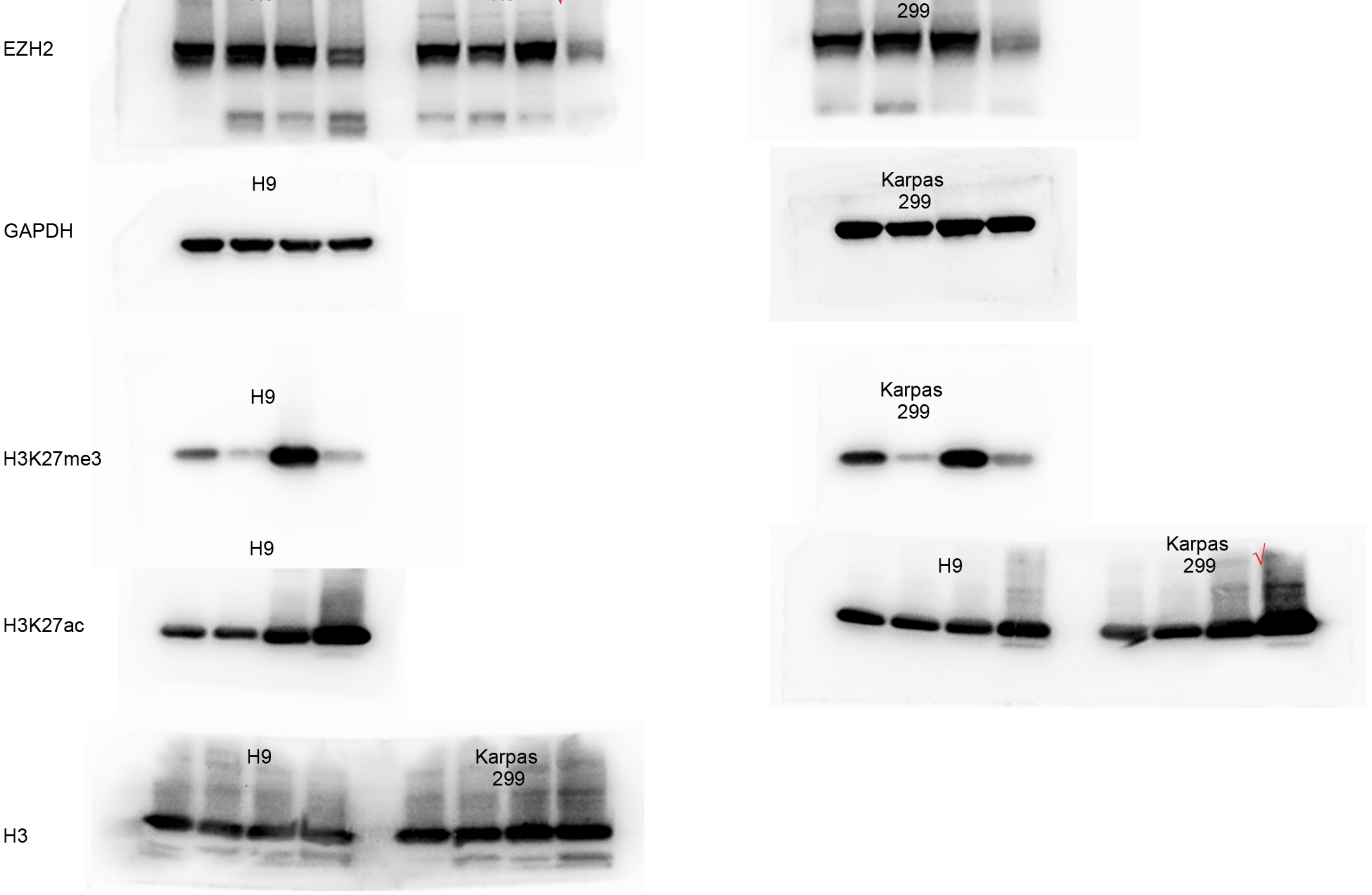

Fig. 4c

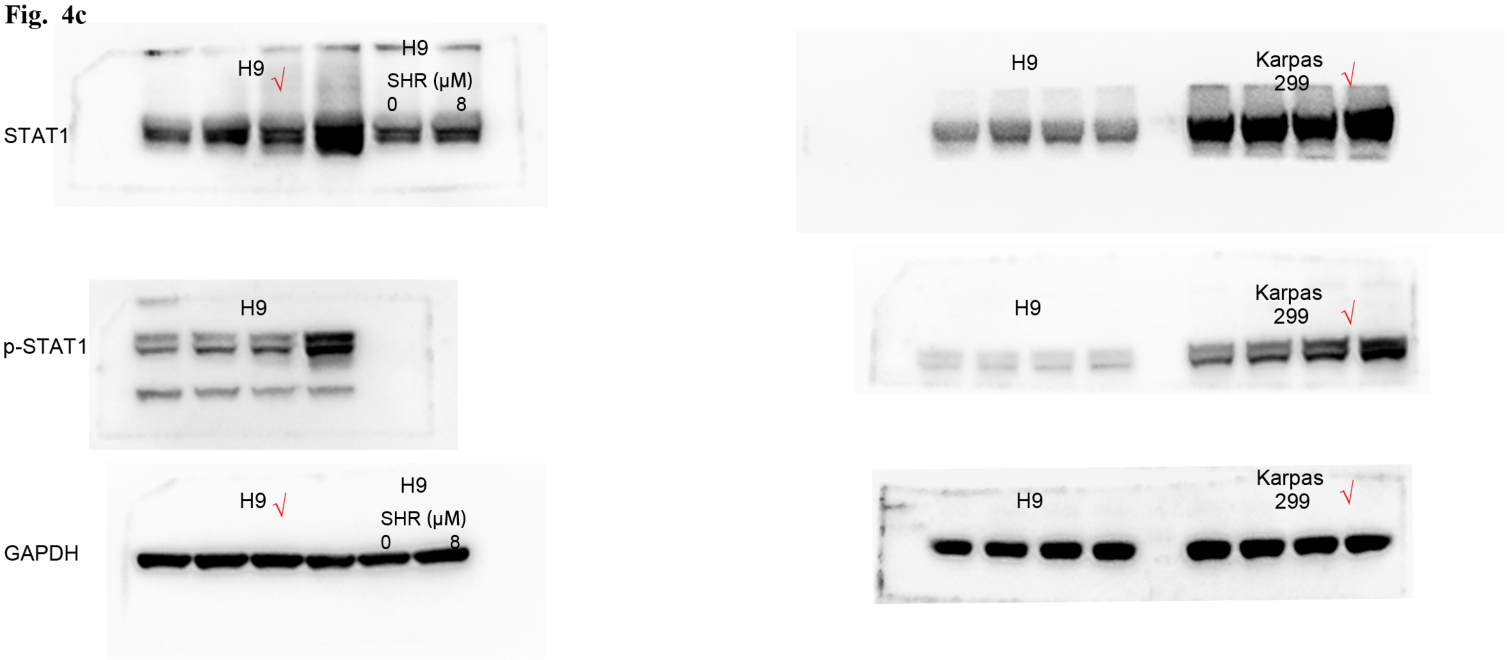

Fig. 4e

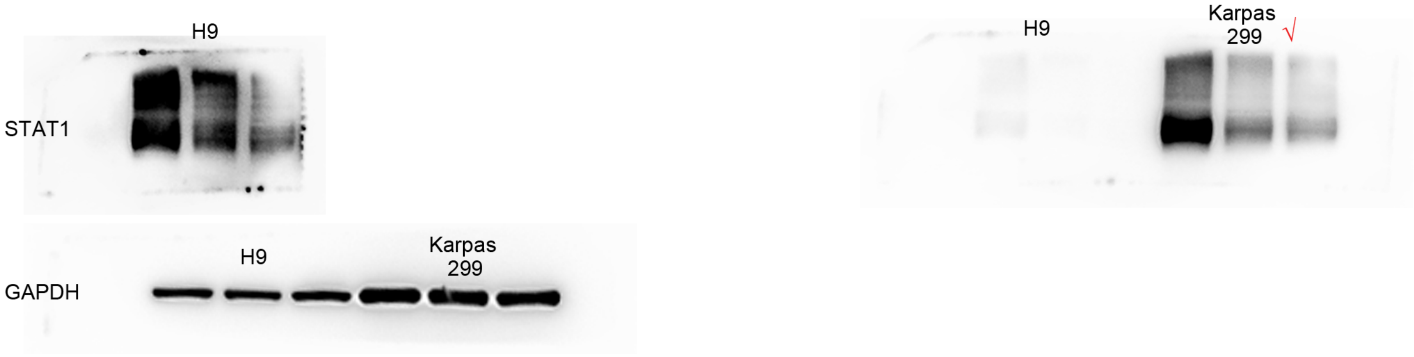

Fig. 4h

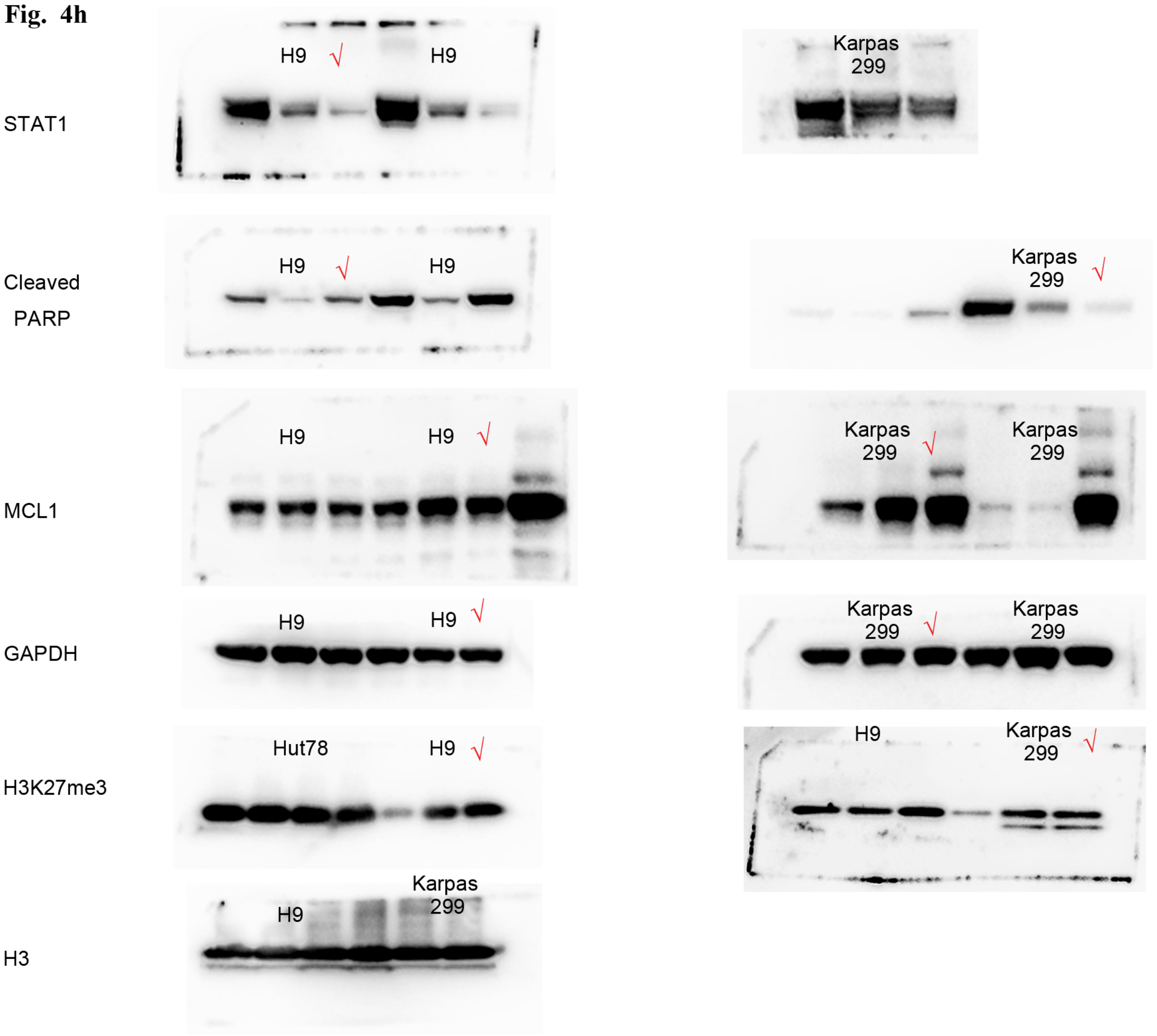
STAT1
Cleaved PARP
MCL1
GAPDH
H3K27me3
H3
H9
H9
Karpas 299
H9
H9
Karpas 299
H9
H9
Karpas 299
Karpas 299
H9
H9
Karpas 299
Karpas 299
Hut78
H9
H9
Karpas 299
H9
Karpas 299

Fig. 4i

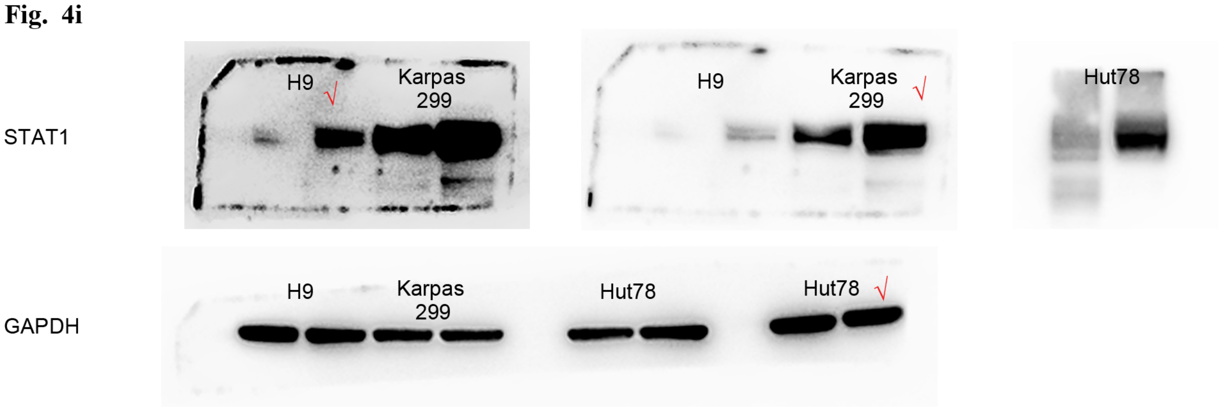
STAT1
GAPDH
H9
Karpas 299
H9
Karpas 299
Hut78
H9
Karpas 299
Hut78
Hut78

Fig. 4n

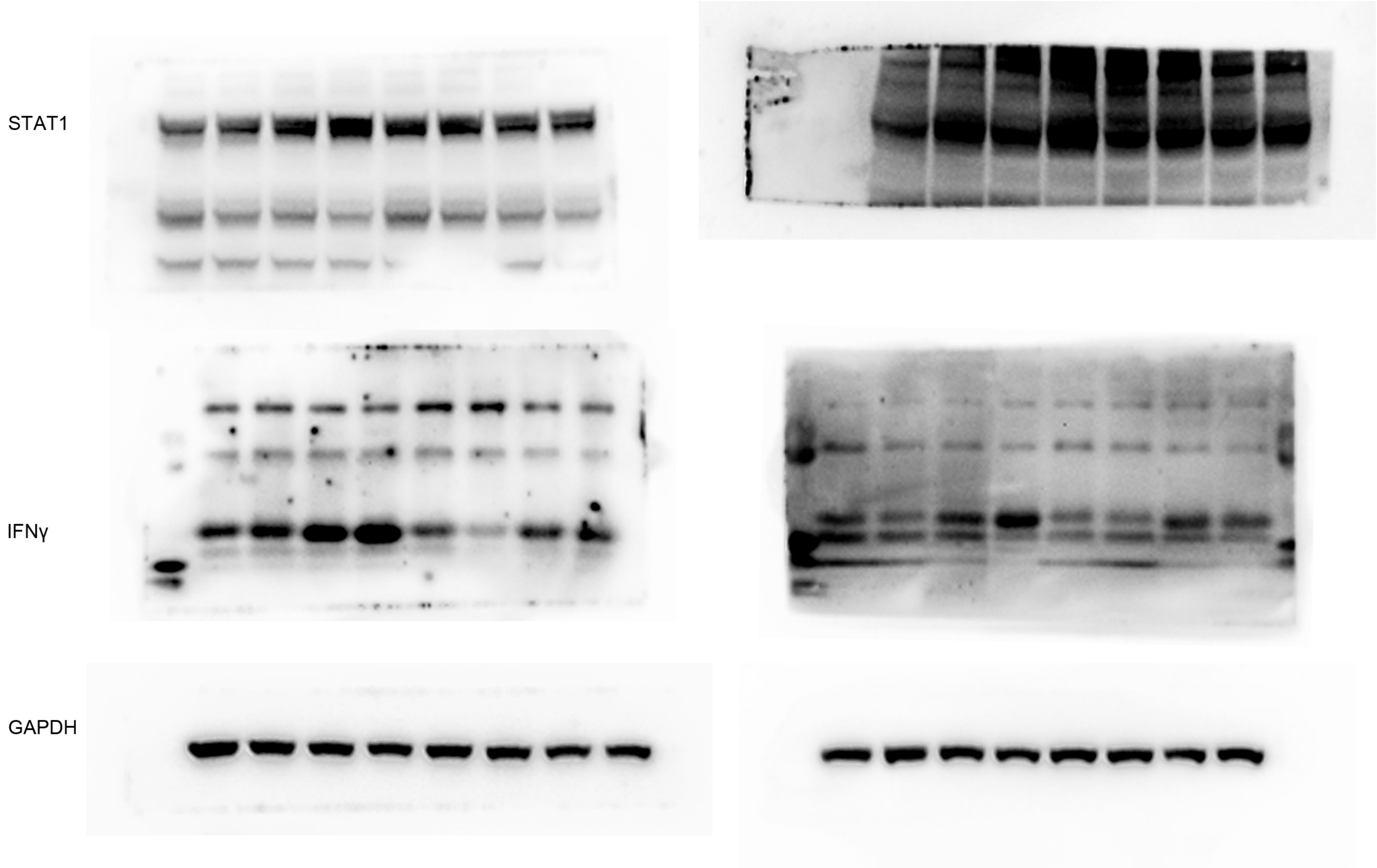

Fig. 5e

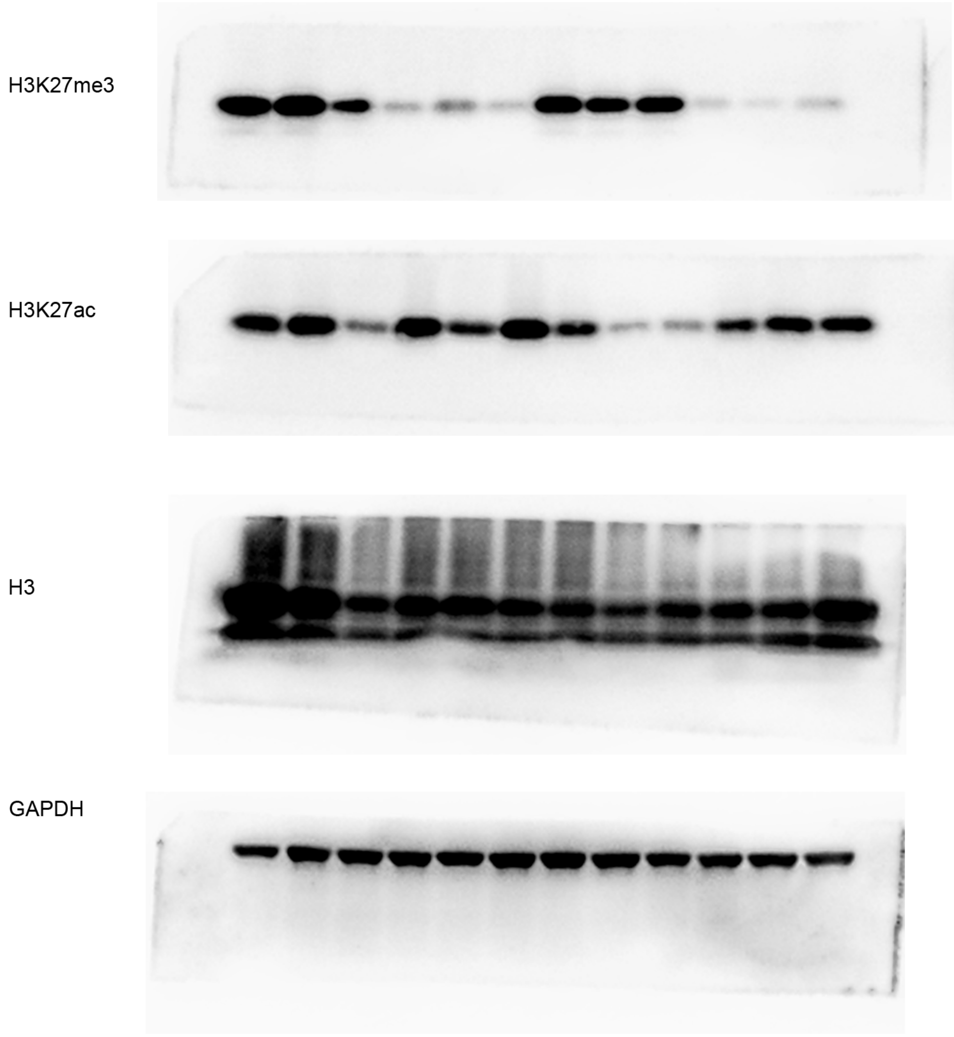

Fig. S1b, d

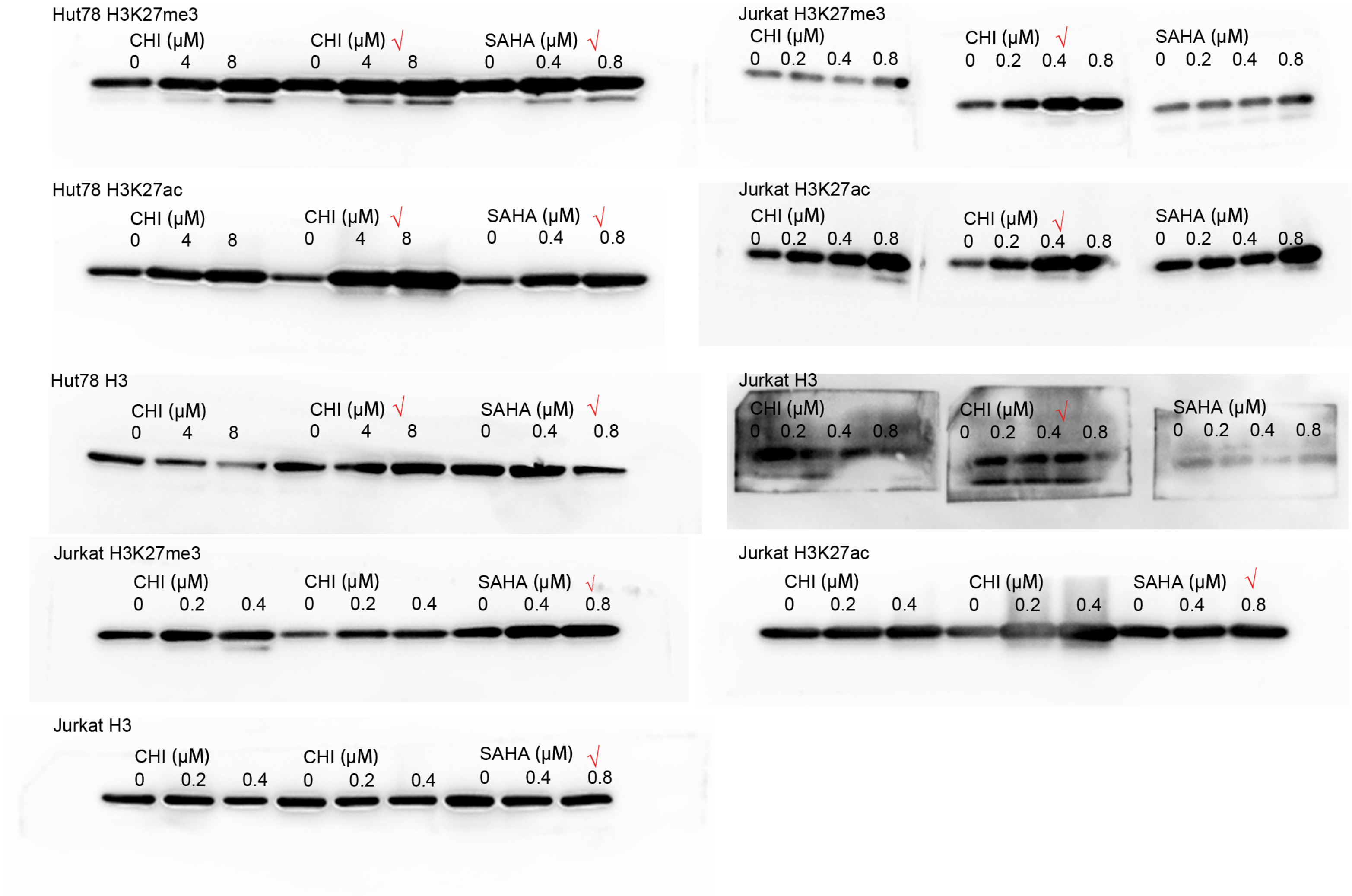

Fig. S1f

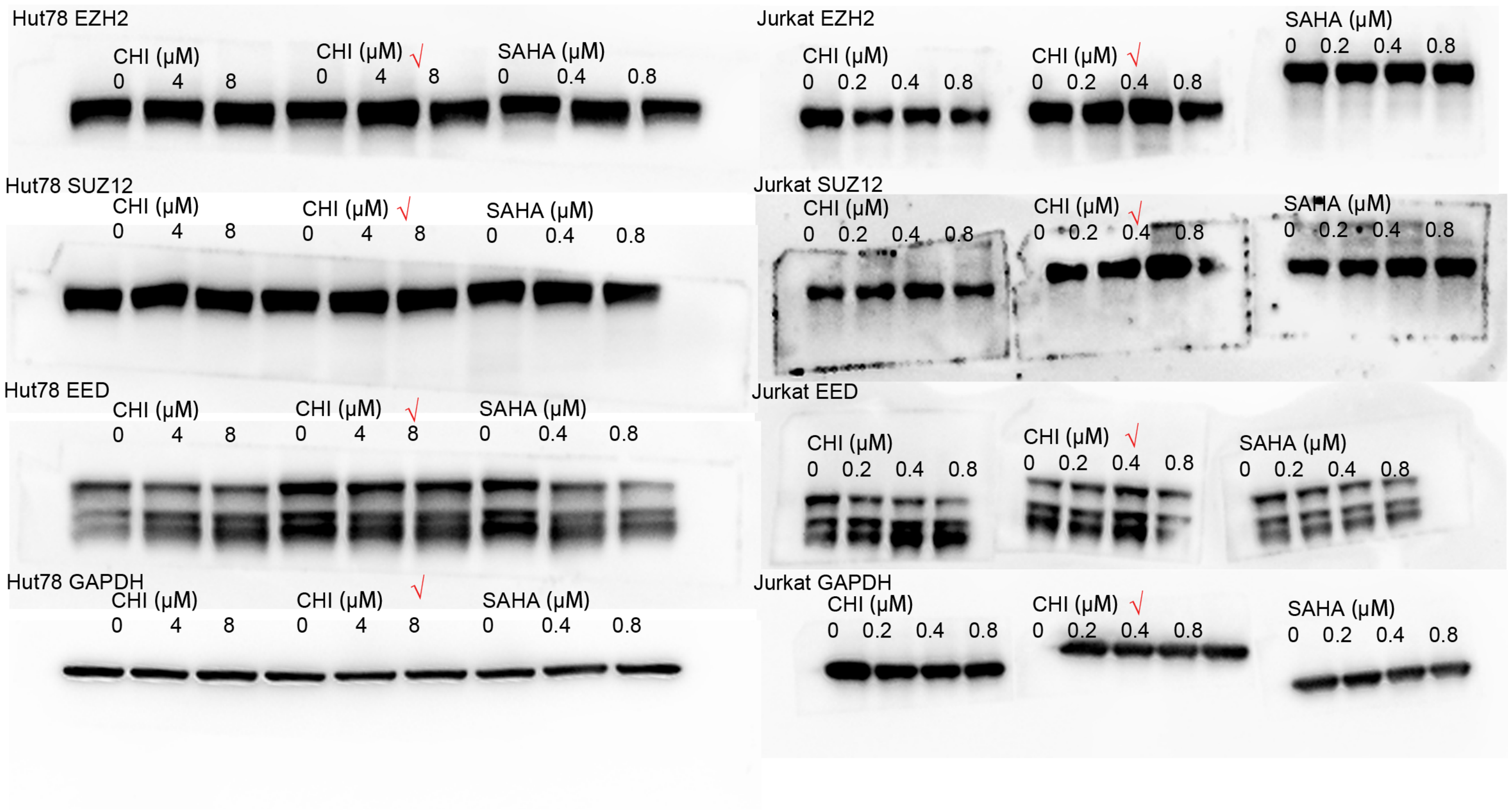

Fig. S2a-b

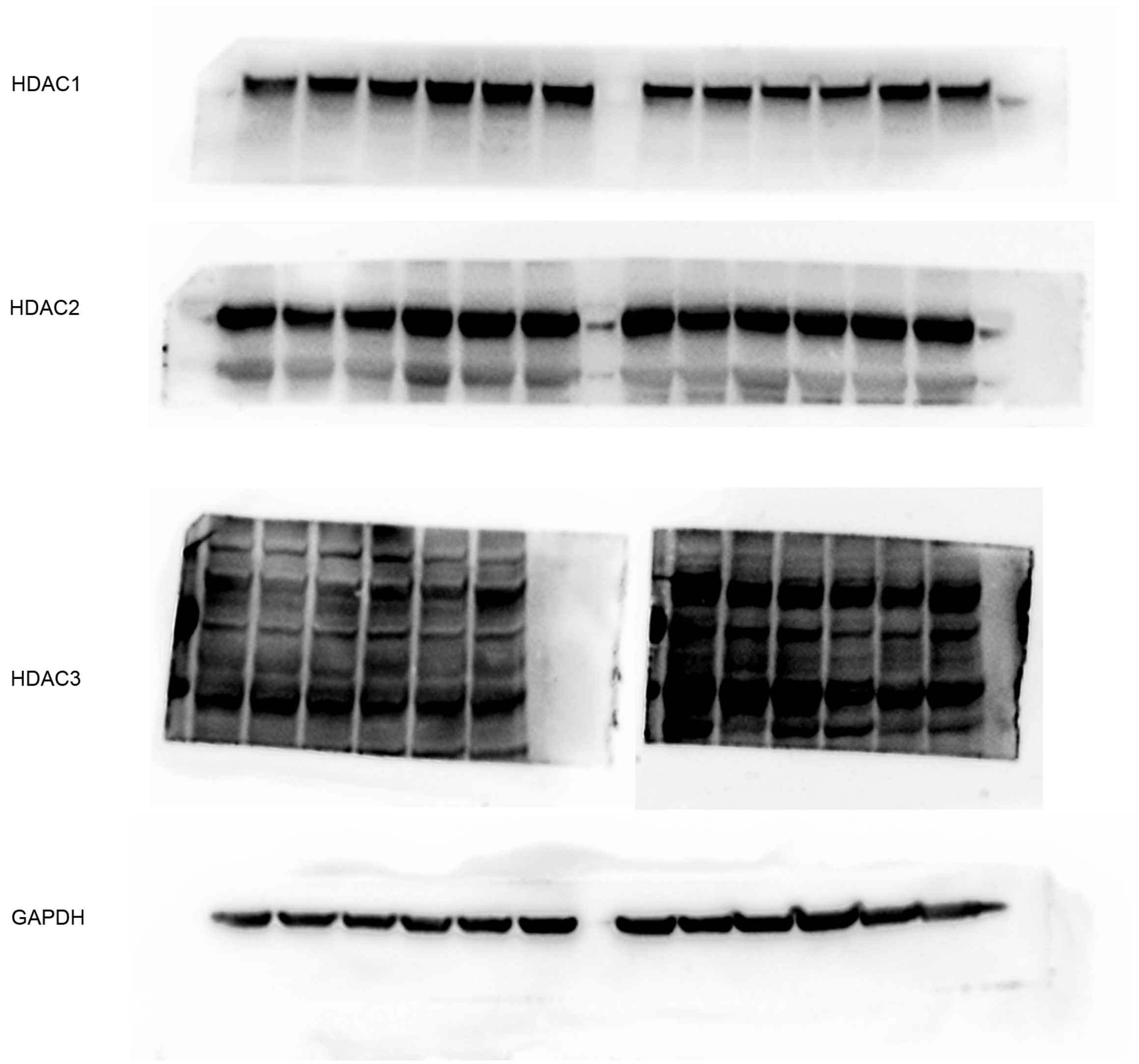

Fig. S4c

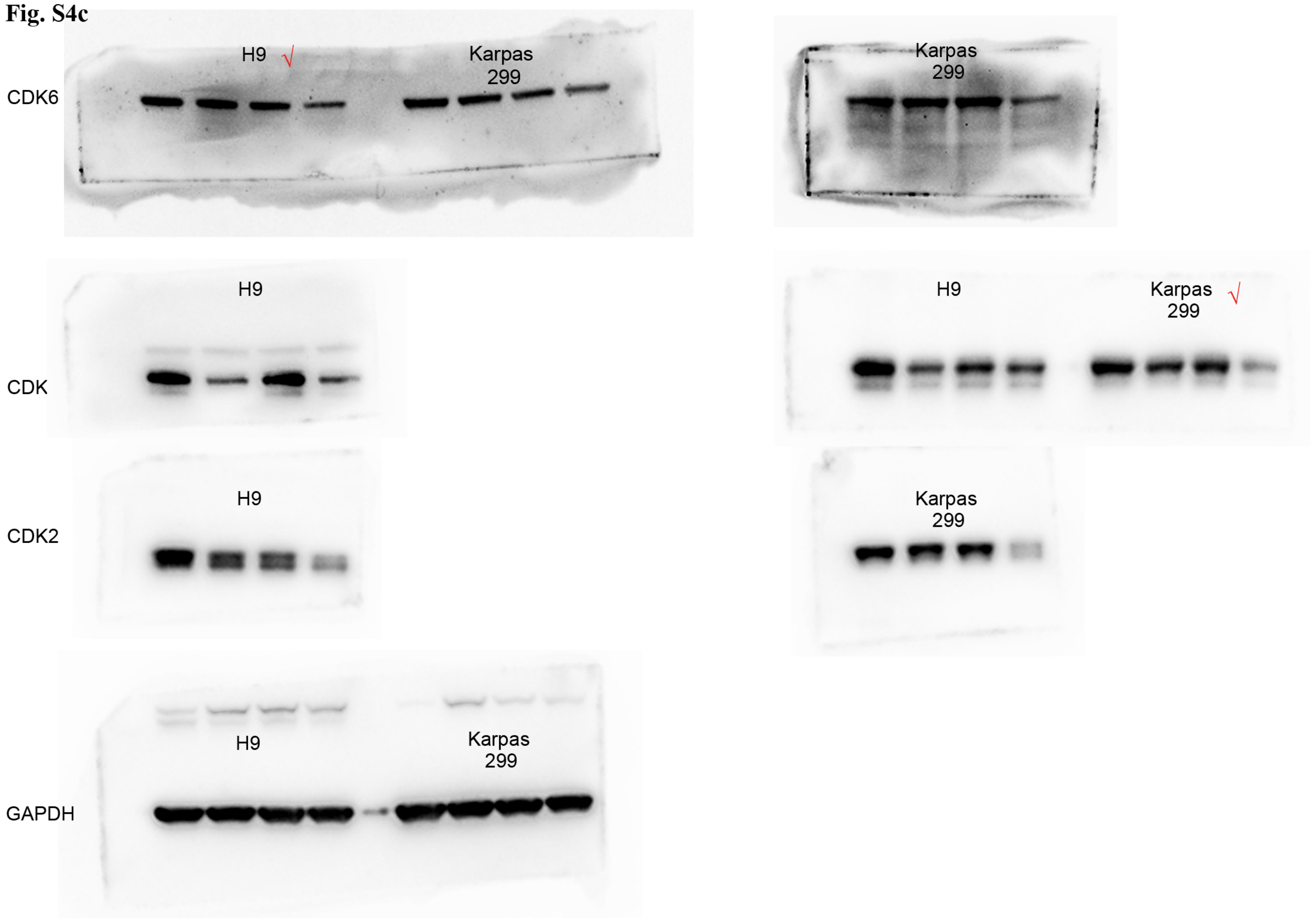

Fig. S4e

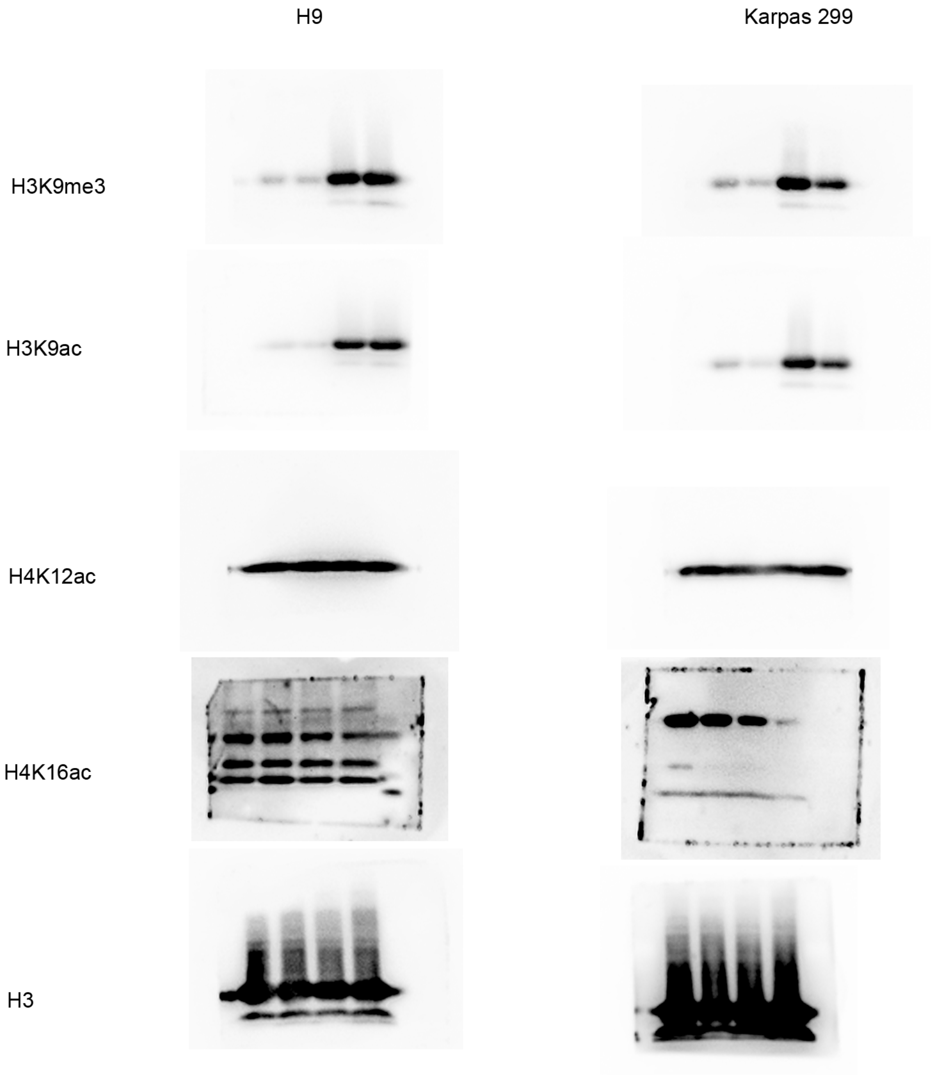

Fig. S5e

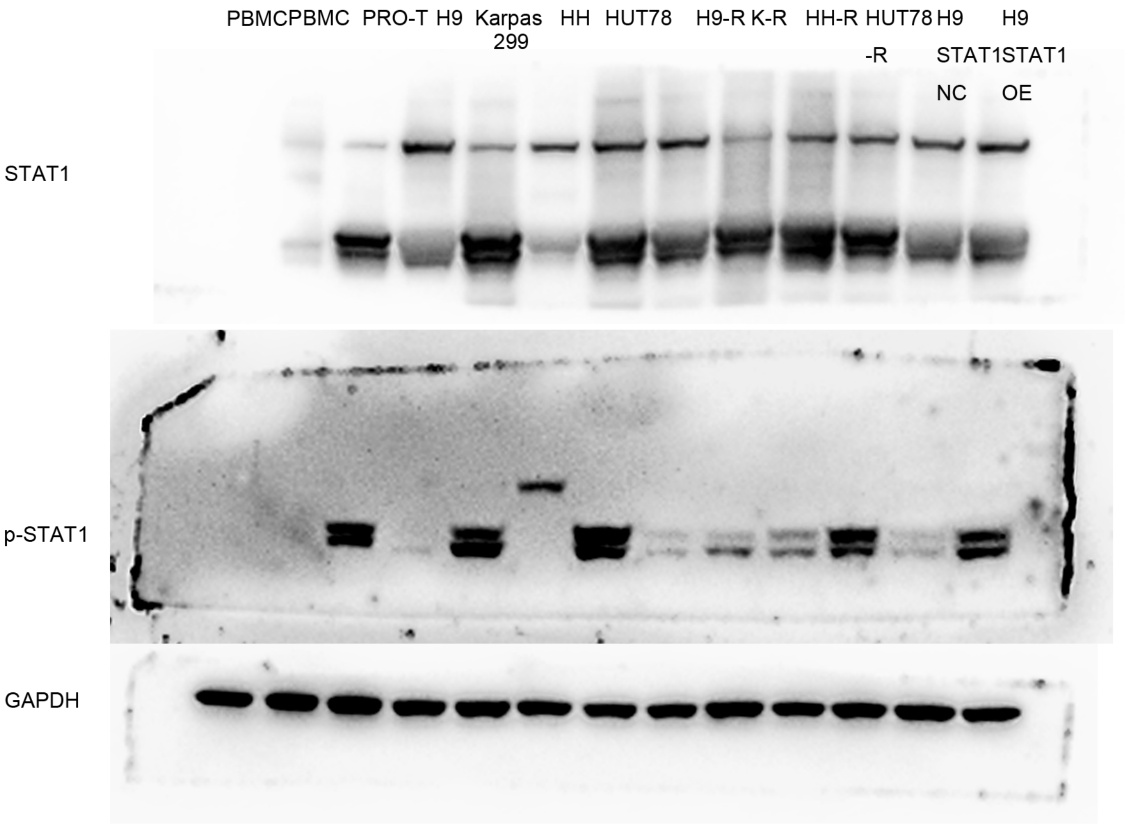

Fig. S6g

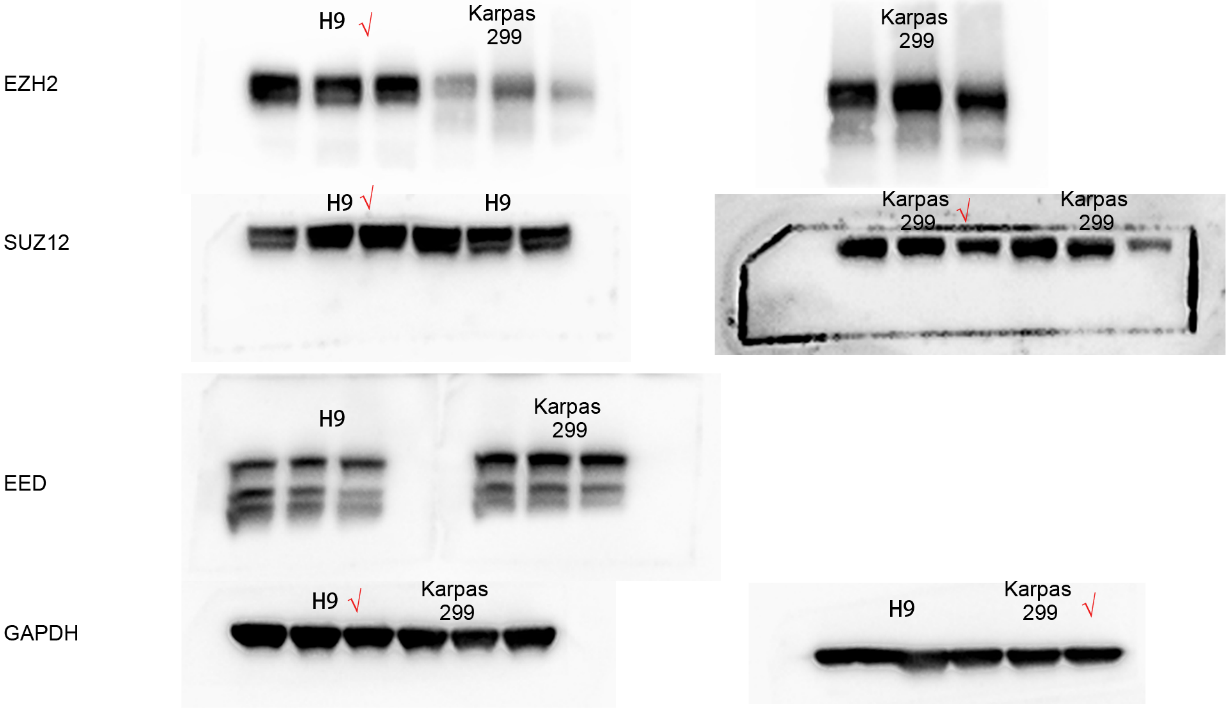

Fig. S6i

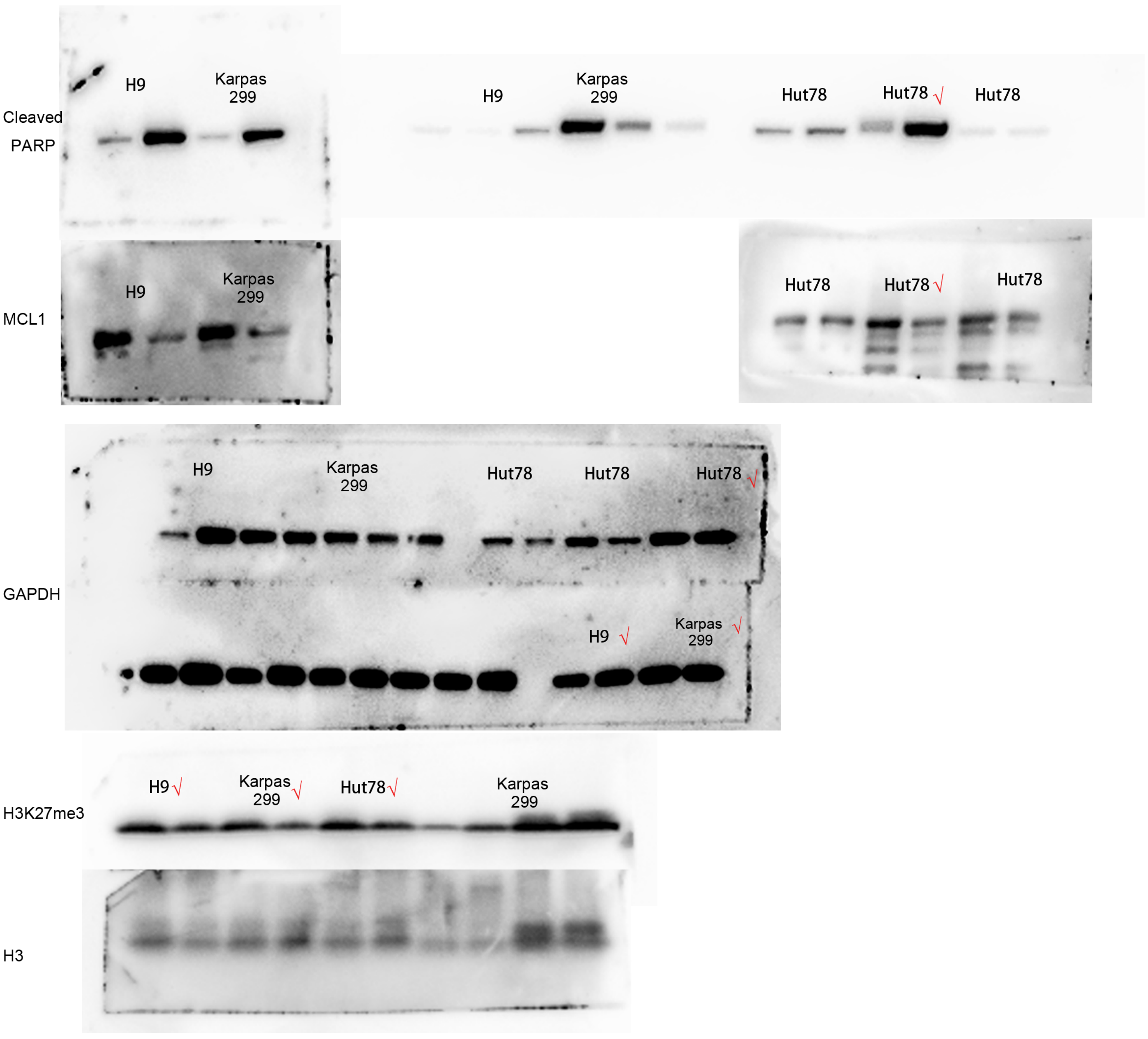

Fig. S8d

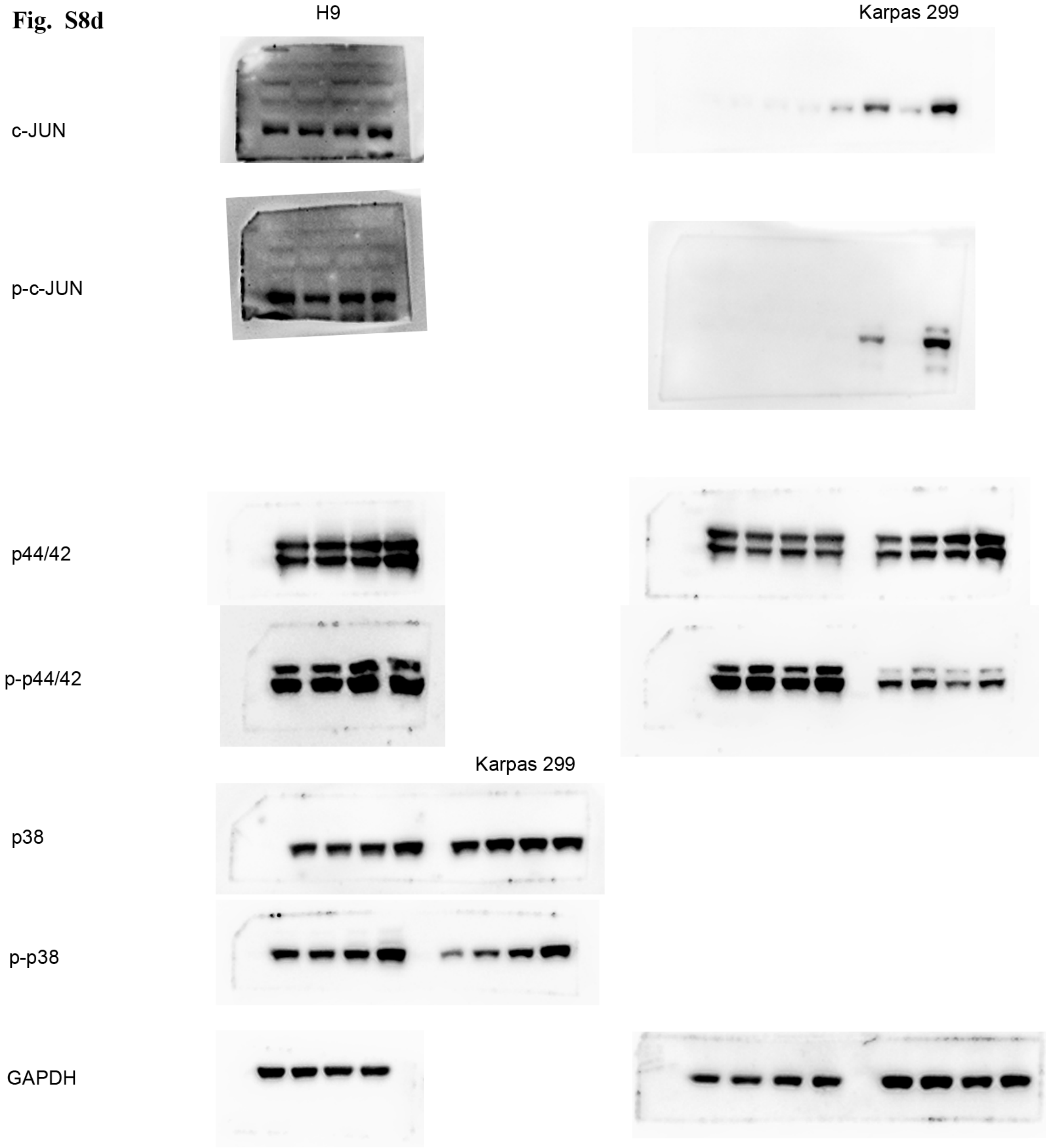
H9
Karpas 299
c-JUN
p-c-JUN
p44/42
p-p44/42
Karpas 299
p38
p-p38
GAPDH

Fig. 1D	EZH2 mRNA Relative expression		
	CT	CHI 0.2μM	CHI 0.4μM
H9	1	1.278956	1.712484
	1	1.112739	1.73018
	1	0.938314	1.470467
	CT	CHI 0.4μM	CHI 0.8μM
Karpas 299	1	1.927287	2.553348
	1	2.306763	2.352766
	1	2.418644	2.52579

Fig. 4D	STAT1 mRNA Relative expression			
	CT	SHR 8μM	CHI 0.2μM	Combo
H9	1	3.454098	1.600817	8.069759
	1	3.801335	2.001352	9.188802
	1	4.137776	2.470395	11.99129
	CT	SHR 20μM	CHI 0.2μM	Combo
Karpas 299	1	0.830516	1.257766	1.633857
	1	0.761166	1.363713	1.577709
	1	0.995928	1.238206	1.951219

Fig. 4O	IFNγ mRNA Relative expression			
	CT	SHR 8μM	CHI 0.2μM	Combo
H9	1	30.04825	2.514058	79.86885
	1	18.88706	4.236538	63.35179
	1	34.76648	3.680463	110.2081
	CT	SHR 20μM	CHI 0.2μM	Combo
Karpas 299	1	1.739113	11.33741	16.51371
	1	1.517144	10.40888	16.0685
	1	3.475382	11.96632	19.88327

Fig. S2C	SUZ12 mRNA Relative expression		
	CT	CHI 0.2μM	CHI 0.4μM
H9	1	0.753609	0.684759
	1	1.28269	0.658284
	1	1.32454	0.728939
	CT	CHI 0.4μM	CHI 0.8μM
Karpas 299	1	1.137665	1.525235
	1	1.170039	1.345822
	1	1.104308	1.302103

Fig. S2C	EED mRNA Relative expression		
	CT	CHI 0.2μM	CHI 0.4μM
H9	1	1.007123	0.822938
	1	0.696753	0.820094
	1	0.855222	0.919552
	CT	CHI 0.4μM	CHI 0.8μM
Karpas 299	1	1.979762	2.677161
	1	2.269831	2.598446
	1	2.385156	2.789025

Fig. S6B	STAT1 mRNA Relative expression		
	NC	shSTAT1#1	shSTAT1#2
H9	1	0.270364	0.275627
	1	0.215966	0.103664
	1	0.224271	0.087767
	NC	shSTAT1#1	shSTAT1#2
Karpas 299	1	0.319384	0.263183
	1	0.43592	0.238817
	1	0.454622	0.242479
